# In Silico and In Vitro Evaluation of the Mechanism of Action of Three VX809-Based Hybrid Derivatives as Correctors of the F508del CFTR Protein

**DOI:** 10.3390/ph16121702

**Published:** 2023-12-08

**Authors:** Debora Baroni, Naomi Scarano, Alessandra Ludovico, Chiara Brandas, Alice Parodi, Dario Lunaccio, Paola Fossa, Oscar Moran, Elena Cichero, Enrico Millo

**Affiliations:** 1 Istituto di Biofisica, Consiglio Nazionale delle Ricerche (CNR), Via De Marini, 6, 16149 Genova, Italy; aleludo89@gmail.com (A.L.); oscar.moran@cnr.it (O.M.); 2 Department of Pharmacy, Section of Medicinal Chemistry, School of Medical and Pharmaceutical Sciences, University of Genova, Viale Benedetto XV, 3, 16132 Genoa, Italy; naomi.scarano@edu.unige.it (N.S.); paola.fossa@unige.it (P.F.); 3 Department of Experimental Medicine, Section of Biochemistry, University of Genoa, Viale Benedetto XV 1, 16132 Genova, Italy; alice.parodi1994@gmail.com (A.P.); dario.lunaccio@edu.unige.it (D.L.); enrico.millo@unige.it (E.M.)

**Keywords:** CFTR, CFTR correctors, VX445, VX661, molecular docking, YFP functional assay, biochemical assays, NBD1

## Abstract

Cystic fibrosis (CF), the most common autosomal recessive fatal genetic disease in the Caucasian population, is caused by mutations in the gene encoding the cystic fibrosis transmembrane conductance regulator (CFTR), an anion channel that regulates salt and water transport across a variety of secretory epithelia. Deletion of phenylalanine at position 508, F508del, the most common CF-causing mutation, destabilises the CFTR protein, causing folding and trafficking defects that lead to a dramatic reduction in its functional expression. Small molecules called correctors have been developed to rescue processing-defective F508del CFTR. We have combined in silico and in vitro approaches to investigate the mechanism of action and potential as CFTR correctors of three hybrid derivatives (**2a**, **7a**, and **7m**) obtained by merging the amino-arylthiazole core with the benzodioxole carboxamide moiety characterising the corrector lumacaftor. Molecular modelling analyses suggested that the three hybrids interact with a putative region located at the MSD1/NBD1 interface. Biochemical analyses confirmed these results, showing that the three molecules affect the expression and stability of the F508del NBD1. Finally, the YFP assay was used to evaluate the influence of the three hybrid derivatives on F508del CFTR function, assessing that their effect is additive to that of the correctors **VX661** and **VX445**. Our study shows that the development and testing of optimised compounds targeting different structural and functional defects of mutant CFTR is the best strategy to provide more effective correctors that could be used alone or in combination as a valuable therapeutic option to treat an even larger cohort of people affected by CF.

## 1. Introduction

The multi-organ disease cystic fibrosis (CF) is caused by mutations in the gene encoding the CF transmembrane conductance regulator (CFTR) protein, a cAMP-regulated chloride and bicarbonate channel. CFTR is mainly expressed on the apical side of secretory epithelia, including the lungs, pancreas, and testis [1,2].

In CF airways, defects in anion transport by CFTR protein lead to the formation of thick and sticky mucus that accumulates on the apical surface of the epithelium.

Chronic inflammation, persistent untreatable bacterial colonisation, and recurrent chest infections are characteristic features of the disease, eventually leading to progressive lung destruction and respiratory failure [3]. Defective CFTR function is also associated with intestinal problems, including bowel obstruction and an exaggerated immune response to food antigens [4].

CFTR (ABCC7) is a member of the ATP-binding cassette (ABC) transporter superfamily. It is a modular protein composed of five distinct domains: two transmembrane-spanning domains, MSD1 and MSD2; two nucleotide-binding domains, NBD1 and NBD2; and a regulatory domain, RD, unique to this multi-domain protein [5]. To date, more than 2000 variants have been reported [6] and classified into six groups according to their molecular defects: impaired protein synthesis (class I), defective processing and trafficking (class II), gating abnormalities (class III), reduced single-channel conductance (class IV), reduced protein synthesis (class V), and reduced cell surface stability (class VI) [7,8].

However, disease-associated CFTR mutations tend to have multiple biochemical defects. For example, the most common CFTR mutation, F508del, is a class II mutation, although the few F508del CFTR proteins that successfully reach the cell membrane exhibit gating defects (class III) and reduced plasma membrane stability (class VI) [9,10].

Localised in NBD1, the F508del mutation reduces the intrinsic stability of this domain and disrupts interactions between NBD1 and NBD2, as well as those between NBD1 and the membrane-spanning domains (MSDs) [11,12,13,14,15].

Indeed, the crystal structures of isolated WT and F508del NBD1 in solution showed little conformational differences, except for some changes in the local surface topography in the vicinity of the deleted F508 residue [16]. Surprisingly, three-dimensional modelling of NBD1 reveals that the F508 residue is not located in the active site of NBD1 but is exposed on the domain surface, where it plays a role in the interaction with NBD2 and the MSDs [16,17,18]. Consistent with these findings, limited proteolysis analysis of full-length F508del CFTR reveals a more pronounced defect in domain–domain interaction than in F508del NBD1 conformation [19].

Initially, the treatment of CF aimed to prevent or alleviate the manifestations of the disease, mainly recurrent lung infections and exocrine pancreatic failure, by means of symptomatic therapies (antibiotics, mucolytics, bronchodilators, nutritional supplements to prevent malnutrition, etc.) rather than correcting the basic defects of CFTR [20,21]. The development of small molecules called CFTR modulators, which target the underlying molecular defects caused by mutations in the CFTR gene, has revolutionised the therapeutic approach to cystic fibrosis [22,23,24].

According to their mechanism of action, CFTR modulators can be categorised into four groups. Potentiators are drugs that restore defective CFTR gating and are effective in patients with class III and IV mutations. Correctors are small-molecule drugs that facilitate the folding, processing, and trafficking of the CFTR protein to the cell membrane. They are effective in patients with class II mutations. Amplifiers are molecules that increase the expression of CFTR mRNA, while read-through agents are drugs that promote ribosomal over-reading of the premature termination codons that characterise class I mutations. Finally, stabilisers are investigational drugs that increase the stability of the CFTR protein on the plasma membrane [25].

Three class of correctors have been identified based on their mechanism of action, namely redundancy or additivity: (i) Type I correctors targeting the interfaces of NBD1/MSD1 and NBD1/MSD2, (ii) Type II correctors acting on NBD2 and/or its interfaces, and (iii) Type III correctors affecting the folding and stability of NBD1 [26,27].

**Figure 1:**
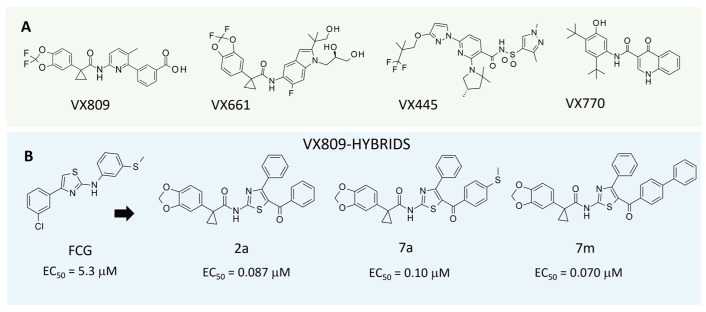
Scheme of the chemical structure featured by known CFTR modulators that have reached the market (**VX809**, **VX661**, **VX445**, **VX770**). (**A**) The chemical structure and the corrector half-maximal effective concentration featured by hybrid derivatives investigated herein are reported (**B**).

It is widely accepted that the administration of combinations of correctors with different mechanisms of action and different binding sites on the CFTR protein would provide a more relevant rescue of the defects associated with disease-causing CFTR mutations than the use of a single therapeutic agent [26,28]. Indeed, with the exception of the potentiator, Kalydeco (ivacaftor, **VX770**), all the FDA- and EMA-approved CFTR drugs targeting the underlying genetic cause of CF currently on the market consist of a combination of the potentiator ivacaftor (**VX770**) and one or two correctors: Orkambi, made up of **VX770** and the corrector lumacaftor (**VX809**); Symdeko, a combination of **VX770** and the corrector tezacaftor (**VX661**); and the latest Trikafta/Kaftrio, which combines the **VX770** with the correctors tezacaftor (**VX661**) and elexacaftor (**VX445**) (see Figure 1) [29,30,31,32].

The advent of CFTR modulators has undoubtedly changed the clinical course of CF, leading to significant improvements in the lives of a large proportion of people with CF, whose life expectancy has increased from 5 years in 1960 to over 50 years. However, the use of these CFTR drugs is limited to certain mutations, and almost 15% of people with CF remain without any CFTR-targeted therapy [33,34]. The encouraging results obtained so far have motivated further efforts to discover new correctors to be used alone or in combination with already existing therapeutics to treat a wider cohort of people with CF. In this context, in recent years, we have designed and synthesised several amino-arylthiazoles (AATs) capable of acting as CFTR potentiators and/or correctors, some of which demonstrated synergistic or additive effects with **VX809** [35,36,37].

We have also applied different biochemical approaches to elucidate the mechanism of action of one of these thiazole derivatives, **FCG** (see Figure 1), demonstrating that NBD2 is required for its activity as a corrector of the basic defect of F508del CFTR [38]. Specifically, our western blot analysis of singly expressed CFTR domains revealed that FCG improved either the total expression and the maturation of NBD2 without affecting the expression of the other domains. Additionally, the cycloheximide chase assay pointed out that FCG significantly increases the stability of NBD2.

More recently, we have synthesised and developed new derivatives that act as CFTR correctors by merging the AAT core with the benzodioxole carboxamide moiety that characterises the corrector **VX809** (now called hybrids [37,38,39]). In particular, we designed a first series of hybrids bearing the benzoyl group at the thiazole position 5. This kind of substitution was predicted as advantageous in our previous quantitative structure-activity relationship (QSAR) studies [37]. Among these derivatives, compound **2a** proved to be the most active (EC_50_ = 0.087 µM) (Figure 1) [36]. Subsequently, other compounds were derived by applying different structural substitutions to the aforementioned benzoyl group of **2a**, leading to compounds such as compounds **7a** and **7m** (Figure 1), endowed with comparable EC_50_ values (Figure 1), or by pursuing concomitant structural modifications at the thiazole positions 4 and 5 [37,38,39].

To guide a future feasible and reliable rational design process of new derivatives, in this study, we combined computational analyses with different biochemical approaches to identify the putative mechanism of action of **2a**, **7a**, and **7m**, the most promising hybrids of our in-house derivative library. The results are expected to provide a feasible and reliable protocol for the rational design of new and more effective thiazole-based modulators for the treatment of CF.

## 2. Results

### 2.1. Computational Studies

#### 2.1.1. In Silico Structure-Based Studies Assessment

For many years, the lack of a high-resolution CFTR structure represented a significant bottleneck in the search for novel CFTR modulators. Recently, progress was made in cryo-Electron Microscopy (cryo-EM) to pave the way for insights into the complete CFTR structure [5]. From 2017, a number of experimental data about the wild-type and F508del CFTR protein became available, in the presence or not of different modulators (see Table 1).

Herein, to identify in silico the CFTR most probable binding regions affected by the action of the compounds investigated in this study (**2a**, **7a**, **7m**; see chemical structure in Figure 1), the structural information associated with the PDB codes 8EIG, 8EIO, and 8EIQ [40] for F508del CFTR in the presence at of corrector **VX445** alone (8EIG) or in combination with one or two other CFTR modulators (8EIO and 8EIQ) was used. Indeed, this information were endowed with: (i) comparable or better resolution values than the previous ones reported from 2019 to 2022 (6O2P, 7SV7, 7SVD, 7SVR), (ii) key data about the binding mode featured by different classes of CFTR correctors, such as **VX-445**, **VX661**, and **VX809**, (iii) recurrent presence of **VX445** in all of them to preliminarily explore the putative stability of the CFTR-**VX445** complex, in the presence or not of further modulators.

**Figure 2:**
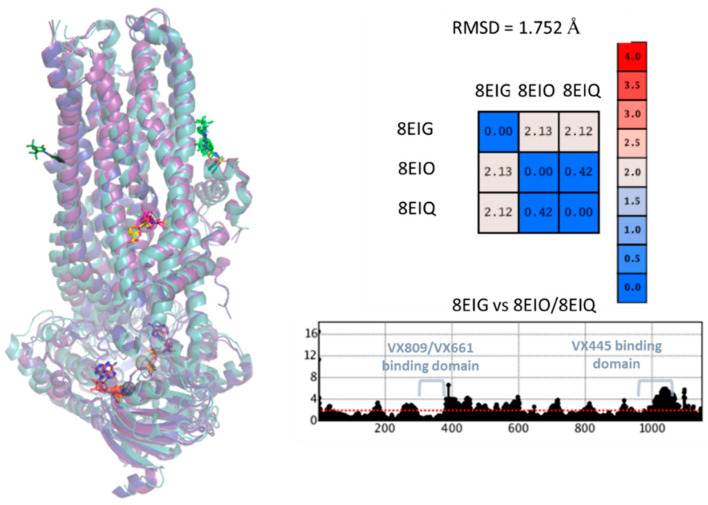
Superimposition of the 8EIG (ribbon in light cyan), 8EIO (ribbon in blue), and 8EIQ (ribbon in violet) experimental data (left). RMSD values as obtained by the superimposition of 8EIG, 8EIO, and 8EIQ are shown, based on the carbon atom alignment, in tandem with the overall RMSD variation trend.

Figure 2 shows the superimposition of the F508del CFTR structures of the 8EIG, 8EIO, and 8EIQ PDB files and the position of their co-crystallised ligands. In the presence of additional modulators, a modest increase in CFTR flexibility was observed compared to that of CFTR in the presence of **VX445** alone. Indeed, as shown by the table in Figure 2, the calculated carbon atom root means square deviation (CA RMSD) values of the distances between the 8EIG pair and 8EIO/8EIQ (CA RMSD = 2.12–2.13 Å) were higher than those observed between the 8EIO and 8EIQ data (CA RMSD = 0.42 Å).

Interestingly, when the three PBD structures were considered separately, a different positioning of the protein cavity surrounding compound **VX445** was observed. This is probably due to the fact that the 8EIG structure included only the corrector **VX445**, whereas the 8EIO and 8EIQ PDBs also included the CFTR modulators **VX809**, **VX661**, and **VX770**. In fact, the effect, played out in terms of RMSD variations along the whole protein, revealed much more protein flexibility around the **VX445** cleft than near the **VX809**/**VX661** binding pocket (Figure 2). These results support the hypothesis of an allosteric behaviour of class I CFTR correctors (**VX809** and **VX661**) with respect to the binding site of **VX445**, which was found to be located on the solvent-exposed surface area of the protein [44].

**Figure 3:**
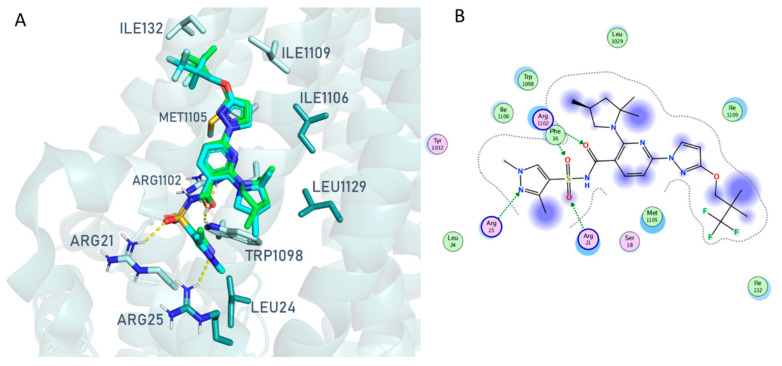
Docking pose of the modulator, **VX445** (C atom; green), at the 8EIG PDB code, with respect to experimental positioning (C atom; cyan). (**A**) Ligplot as a schematic representation of the main protein-ligand contacts is reported. (**B**) Polar and hydrophobic residues are coloured in pink and green, respectively. The ligand portion to be projected outside the protein surface is highlighted in blue.

Accordingly, the **VX445** binding positioning on the CFTR surface, as well as the protein-ligand interactions, were slightly different from the 8EIG to the 8EIO, 8EIQ data. In fact, the 8EIG PDB code suggested key H-bonds involving the dimethyl-substituted imidazole group H-bonded and Arg21, Arg25. The CF_3_-alkyl group and the pyrrolidine ring were projected outside the surface protein (Figure 3).

Conversely, the pyrrolidine and imidazole substituents at position 2 and 5 of the pyridine ring of **VX445** rotated much closer to the folded surface of CFTR in the presence of the co-crystallised class I modulators, **VX809** and **VX661**, as shown in 8EIO and 8EIQ rather than in 8EIG. This allowed the compound to move the main pyridine ring and the tethered sulfonamide group in proximity to Arg21, Trp1098, and Arg1102, revealing additional H-bonds and cation-π stacking within the 8EIO/8EIQ experimental data. In particular, based on the interactions mentioned, the presence of **VX809** rather than **VX661** proved to be more efficient in stabilizing the positioning of **VX445** on the protein surface, thus gaining more contact with the biological target. In any case, the protein cavity containing corrector **VX445** is the same in all PDB codes and is referred to here as pocket A (Figure S1).

Regarding the specific binding behaviour of the CFTR class I correctors, the co-crystallised pose of **VX809** and CFTR within the 8EIO PDB code experienced H-bonds involving the terminal carboxylic group and the CFTR residues, Lys68 and Arg74, with the benzodioxole portion involved in π-π stacking with Phe81 and Trp361 (Figure S2).

This positioning allowed the compound to fit properly into the CFTR cavity on the channel surface. Similarly, the experimental data on **VX661** showed that the π-π stacking between the benzodioxole group and the previously mentioned Phe81 and Trp361 was maintained, while the substituents at indole positions 1 and 2 were H-bonded to Asn71 and Arg74, respectively (Figure S3). This behaviour is expected to ensure the correct CFTR-ligand binding mode, albeit at the expense of a salt-bridge with Lys68, as previously described for **VX809**. However, the binding cavity of **VX809** and **VX661** is the same in both the PDB codes and is designated as pocket B. This positioning allowed the compound to fit properly into the CFTR cavity at the channel surface.

Notably, mutual allosteric behaviour could be noticed between **VX445** and **VX809**/**VX661**, being the last two CFTR modulators better stabilised at the protein cavity when combined with **VX445** rather than alone, based on the aforementioned experimental poses (Figures S2 and S3). As shown in the PDB codes, 7SVD and 7SV7, **VX809** and **VX661** featured limited H-bond contacts or polar interactions with the CFTR protein when they are the only bound modulators to CFTR (see Figures S4 and S5, respectively).

#### 2.1.2. Molecular Docking Studies of Hybrids **2a**, **7a**, **7m**

**Figure 4:**
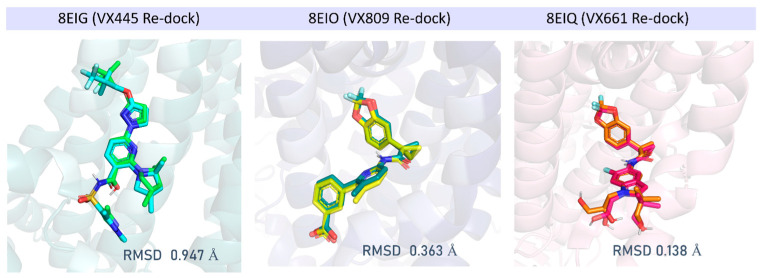
Re-docking performances evaluated as RMSD (Å) of the docking pose with respect to the corresponding experimental data, referring to 8EIG, 8EIO, and 8EIQ respectively. RMSD values have been calculated via DockRMSD [47].

Molecular docking studies were then performed with the **VX809** hybrid derivatives, **2a**, **7a**, and **7m**, previously developed by us as promising CFTR correctors, considering the aforementioned PDB codes, 8EIG and 8EIO, 8EIQ [40]. Indeed, these three experimental data allowed us to evaluate in silico the putative hybrid binding ability to the previous **VX445** and **VX809**/**VX661** binding cavities. In order to preliminarily evaluate the reliability of the docking protocol to be used for hybrid docking simulations, we performed a re-docking of the three X-ray protein-ligand co-crystallised complexes following a previously applied procedure [45,46]. As shown in Figure 4, correctors **VX445**, **VX809**, and **VX661** were properly docked to the corresponding CFTR binding site as defined by the experimental data. The MOE Dock tool was used to perform template-based docking calculations (see the experimental section). As ten poses were calculated for each re-docked compound, it was possible to evaluate whether the applied final scoring exploited for the conformer ranking efficiently scores the lowest RMSD-based poses as the top ones.

The MOE S score (affinity dG) was able to correctly select the bio-active pose in accordance with the experimental data in 7/10 cases for each ligand, with the best-scored conformers **VX445** (S = −6.5860 Kcal/mol), **VX661** (S = −9.9823 Kcal/mol), and **VX809** (S = −8.7534 Kcal/mol), as endowed with RMSD values < 1 Å. Details of the scoring function values and of the top ten poses are reported in Tables S1–S3.

On this basis, the same docking protocol was applied to the **VX809** hybrids **2a**, **7a**, and **7m**. The full list of obtained poses and the related scoring functions are shown in Tables S1–S3.

To evaluate in silico the most probable binding site for each compound at the 8EIO, 8EIG, and 8EIQ CFTR-ligand complexes, the following parameters were considered: (i) comparison of the obtained scoring functions for each pocket (A and B), (ii) number of different clusters (CLs) containing only comparable poses, taken as a measure of the reproducibility of the best-scored poses. All this information is reported in Table 2.

According to our calculations, among the known CFTR modulators, the binding of **VX445** (S = −6.5860 Kcal/mol) to CFTR pocket A was predicted as more likely than that of **VX809** (S = −5.4008 Kcal/mol) and **VX661** (S = −5.8536 Kcal/mol), in agreement with the experimental data. Conversely, the ability of CFTR correctors, **VX809** (S = −9.0052 to −8.7534 Kcal/mol) and **VX661** (S = −9.9823 to −9.7202 Kcal/mol), to bind CFTR pocket B was calculated to be more likely than that of **VX445** (S = −7.9867 to −7.3970 Kcal/mol).

**Figure 5:**
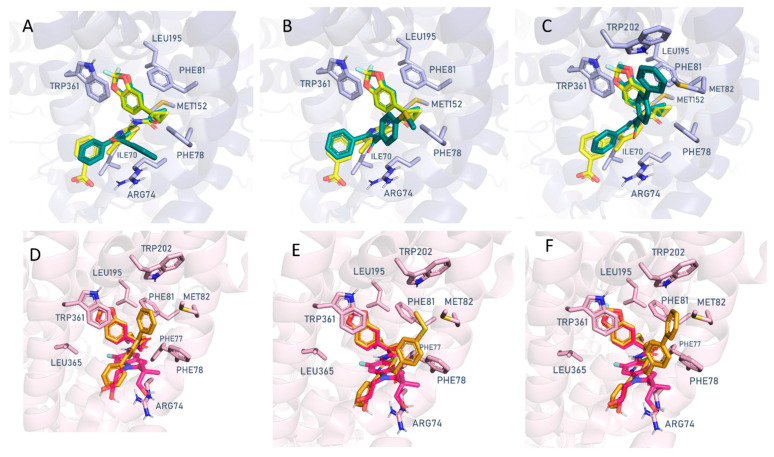
Docking poses of compounds **2a** (C atom; green), (**A**) **7a** (C atom; green), (**B**) and **7m** (C atom; green) (**C**) at the 8EIO/**VX809** binding site (pocket B). The experimental pose of **VX809** is also reported (C atom; yellow). The corresponding poses of **2a** (C atom; orange), (**D**) **7a** (C atom; orange), (**E**) and **7m** (C atom; orange) (**F**) at the 8EIQ/**VX661** binding site (pocket B) are also shown. The experimental pose of **VX661** is also depicted (C atom; magenta).

All the three hybrids, **2a** (EC_50_ = 0.087 µM), **7a** (EC_50_ = 0.10 µM), and **7m** (EC_50_ = 0.070 µM), showed recurrent docking poses at the 8EIO-VX809 binding site (pocket B) with comparable interactions with the CFTR protein (Figure 5A–C). In particular, the corresponding S values ranging from −10.3685 to −7.8760 Kcal/mol agreed with those of the reference compounds, **VX809** (S = −8.7534 Kcal/mol) and **VX661** (S = −9.7202 Kcal/mol), and were also more promising than that of **VX445** (S = −7.3970 Kcal/mol) at pocket B. As shown in Figure 5A,C, both the prototype **2a** (EC_50_ = 0.087 µM) and the optimised **7m** compound (EC_50_ = 0.070 µM) moved the benzoyl-based group towards Arg74 and Phe78, with π-π stacking and cation-π contacts. Further cation-π interactions were also experienced by the phenyl substituent of **2a** and **7m** and the Arg74 residue.

The thiazole ring of the two in-house modulators proved to be the bioisostere of the **VX809** pyridine ring, efficiently superimposing on the reference bioactive conformation. This behaviour was also correctly maintained for the benzodioxol group. Comparable information can be derived for the corresponding docking pose of **7a** (EC_50_ = 0.10 µM) (Figure 5B).

Further molecular docking studies performed on the 8EIQ-**VX-661** binding site (pocket B) confirmed the previously mentioned interactions, despite the different chemo-type used as a template in the molecular docking calculations (see the experimental section for details). In particular, the benzodioxole substituent of **2a** (EC_50_ = 0.087 µM) and of **7m** (EC_50_ = 0.070 µM) were involved in π-π stacking with Trp361, while the benzoyl-based group and the phenyl ring of the two hybrids were projected in proximity to Arg74 and Phe81, as observed for the **VX661** indole-substituted portion (Figure 5D,F).

The final docking pose obtained for the analogue **7a** (EC_50_ = 0.10 µM) is also reported (Figure 5E). The calculated S values of **2a**, **7a**, and **7m**, ranging from −8.8892 to −8.3534 Kcal/mol, support the effectiveness of these compounds as CFTR-targeting ligands, in agreement with those of **VX809** (S = −9.0052 Kcal/mol) and **VX661** (S = −9.9823 Kcal/mol). As a result, pocket B is predicted in silico as the most promising binding cavity for the **2a**, **7a**, and **7m** hybrids. Accordingly, the estimated S values of **2a**, **7a**, and **7m** (S = −5.5739 to −4.4228 Kcal/mol) at the 8EIG-**VX445** site (pocket A) were too high, as were those of the class I correctors **VX809** and **VX661** (S = −5.8536 to −5.4008Kcal/mol), when compared to VX445 (S = −6.5860 Kcal/mol), taken as a pocket A binder.

### 2.2. Biochemical Assays

#### 2.2.1. Cytotoxicity of the Compounds under Analysis

Toxicity data for the tested compounds on HEK-t, FRT, and CFBE41O^−^ cells are presented in Table 3. The Trypan blue survival test yielded TD_50_ values of 27.15 ± 17.88 μM, 11.09 ± 3.22 μM, 7.51 ± 2.22 μM for corrector **VX661**, and 9.60 ± 2.25, 4.29 ± 0.32, 11.75 ± 3.42 μM for corrector **VX445** in HEK-t, FRT, and CFBE41O^−^ cells, respectively. The toxicity of the newly synthetised hybrids was mildly higher than that of correctors **VX661** and **VX445**. In particular, **7m** resulted in the most toxic compound in all the 3 cell lines (TD_50_ values of 1.40 ± 0.20 μM, 5.76 ± 0.89 μM, and 9.78 ± 3.35 μM in HEK-t, FRT, and CFBE41O cells, respectively), while **2a** was the least toxic in HEK (TD_50_ 2.44 ± 1.03 μM) and FRT (TD_50_ 9.24 ± 2.25 μM) cells and **7a** (TD_50_11.97 ± 2.87 μM) in CFBE41O^−^ cells.

The apparently mildly higher TD_50_ values obtained for the three hybrids with respect to the **VX661** and **VX445** correctors were mitigated by their relatively low maximum toxicity values, TD_Max_, in all three cell lines examined. Therefore, considering their cytotoxicity, further experiments were performed with **VX661** and **VX445** at 3 μM and **2a**, **7m**, and **7a** at 2 μM, the concentrations that gave ≥90% cell viability in all three cell lines tested.

#### 2.2.2. Assessing the Impact of Correctors on Full-Length WT and F508del CFTR Functional Expression

**Figure 6:**
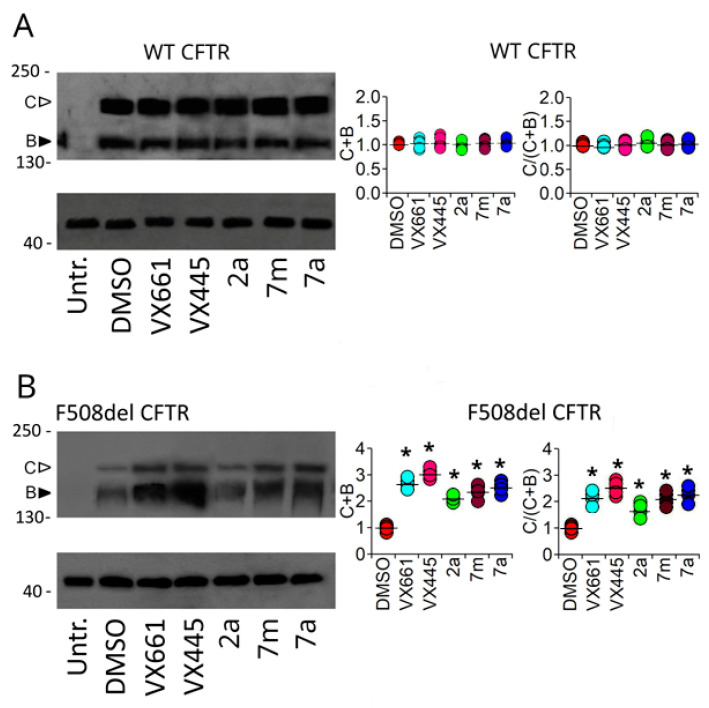
Detection of full-length WT and F508del proteins by Western blot. The upper panels of (**A**) and (**B**) show the expression of WT (**A**) and F508del CFTR (**B**) in whole cell lysates of un-transfected (untr.) and transiently transfected HEK-t cells treated with DMSO (control), **VX661**, **VX445**, **2a**, **7m**, and **7a**. The lower panels of (**A**) and (**B**) show the expression of the housekeeper protein actin in the same samples. The molecular weight of the proteins in the SDS-PAGE molecular weight marker is indicated on the left of each blot. Black and white arrowheads indicate the position of bands B and C, respectively. The graphs in the middle and right of the figure show the quantification of total (sum of bands B and C) and mature (expressed as the ratio of C/(C + B) bands) WT (**A**) or F508del (**B**) CFTR. The expression level of each band was normalised to the level of the actin protein detected in the same samples and expressed relative to the expression level of the control sample. For each condition, the black horizontal lines represent the mean ± standard error of the mean (SEM), while individual measurements are represented by coloured circles. At least 4 independent experiments were performed for each condition. Statistical comparison of data was performed using Dunnett’s post hoc multiple comparison test (all groups versus control). Asterisks indicate statistical significance compared to control, DMSO-treated samples: * *p* < 0.05.

To test the effects of correctors, **VX809**, **VX661**, and newly synthetised hybrids **2a**, **7m**, and **7a** on the expression of full-length WT and F508del CFTR, WT and F508del CFTR transiently transfected HEK-t cells were treated for 24 h with 3 μM **VX661**, 3 μM **VX445**, 2 μM **2a**, 2 μM **7m**, 2 μM **7a**, or DMSO as a control vehicle. Figure 6 shows the immunoblot analysis of obtained whole cell extracts.

The monoclonal antibody MM13-4, raised against the N-terminus of the CFTR protein, detected WT and F508del CFTR isoforms as two electrophoretic bands of approximately 160 and 180 KDa. The lower molecular mass band, band B, corresponds to the immature, core glycosylated, and endoplasmic reticulum (ER)-entrapped form of CFTR, whereas the higher molecular mass band, band C, corresponds to the mature, fully glycosylated, and fully processed isoforms of CFTR, respectively. The most prominent band in the lysates of the WT CFTR-transfected HEK-t cells was the C band (first lane of the upper panel in Figure 6A).

Lysates from HEK-t cells expressing F508del CFTR predominantly showed CFTR band B, consistent with the severe folding and trafficking defects caused by the mutation (first lane of the upper panel of Figure 6B).

Treatment of full-length WT CFTR transfected cells with **VX661**, **VX445**, **2a**, **7m**, and **7a** did not modify either the total expression of WT CFTR, defined as the sum of bands B and C, or the maturation of WT CFTR, expressed as the ratio of the mature, fully glycosylated (band C) to total (B + C bands) isoforms of the protein (plots of Figure 6A).

Conversely, **VX661**, **VX445**, **2a**, **7m**, and **7a** significantly increased F508del total protein and maturation rate (plots of Figure 6B).

Compared to the untreated isoform, the correctors **VX661** and **VX445** increased the total expression of F508del CFTR by almost 2.61 and 3.11 times, and its maturation rate by 2.10 and 2.50 times, respectively. Among the new hybrids, **7a** and **2a** were the most and least effective in rescuing total F508del CFTR expression, increasing F508del total expression by 2.48 and 2.09 times, and its maturation rate by 1.42 and 1.87 times, respectively. The CFTR polypeptide was not detected in non-transfected HEK-t cells.

Table S4 shows the total protein and protein maturation levels for WT and F508del CFTR, normalised to the housekeeping protein actin and to the level of untreated WT or F508del CFTR. Statistical significance determined by Dunnett’s multiple comparison test (all groups versus control) is also shown.

**Figure 7:**
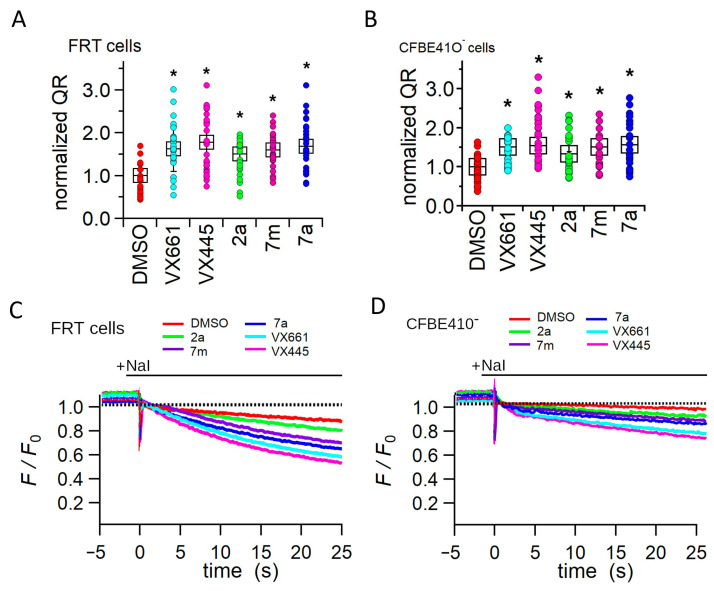
Assessment of F508del CFTR channel function using the YFP assay. F508del CFTR channel activity is expressed as the initial fluorescence quenching rate (QR) of YFP in YFP, and F508del permanently transfected FRT (**A**) and CFBE41O (**B**) cells. Black boxes represent the mean ± standard error of the mean (SEM), while coloured circles represent single measurements (n ≥ 10). The time course of fluorescence decay in FRT (**C**) and CFBE41O^−^ (**D**) cells incubated with DMSO (vehicle control), 3 μM **VX661**, 3 μM **VX445**, 2 μM **2a**, 2 μM **7m**, 2 μM **7a**. For each condition, each measurement (n) was repeated at least 10 times. Asterisks indicate a significant difference (* *p* < 0.05) compared to the control, DMSO-treated samples.

The YFP functional assay was applied to assess whether the increase in protein expression induced by treatment with the compounds under investigation correlated with an increase in F508del CFTR channel activity. To achieve this aim, FRT and CFBE41O cells that permanently express the F508del CFTR and the iodine-sensitive YFP were used. The iodide influx, mediated by F508del channels, was measured as the initial quenching rate (QR) of the fluorescence of the YFP. Figure 7A–D show that treatment with either the correctors **VX661** and **VX445** and hybrids **2a**, **7m**, and **7a** significantly increased the iodide influx in both FRT and CFBE41O cells.

Specifically, in FRT and CFBE41O^−^ cells, **VX661** induced an augment in the QR that was 1.63 and 1.46 times higher than that of the control condition, while **VX445** increased the QR by 1.78 and 1.55 times, respectively (Figure 7). The hybrids also caused a significant increase in iodide influx in FRT and CFBE41O^−^ cells. In both cell preparations, the effect of the hybrids on QR was **7a** > **7m** > **2a** (Figure 7).

#### 2.2.3. Assessing the Impact of Correctors on CFTR Single Domain Expression

**Figure 8:**
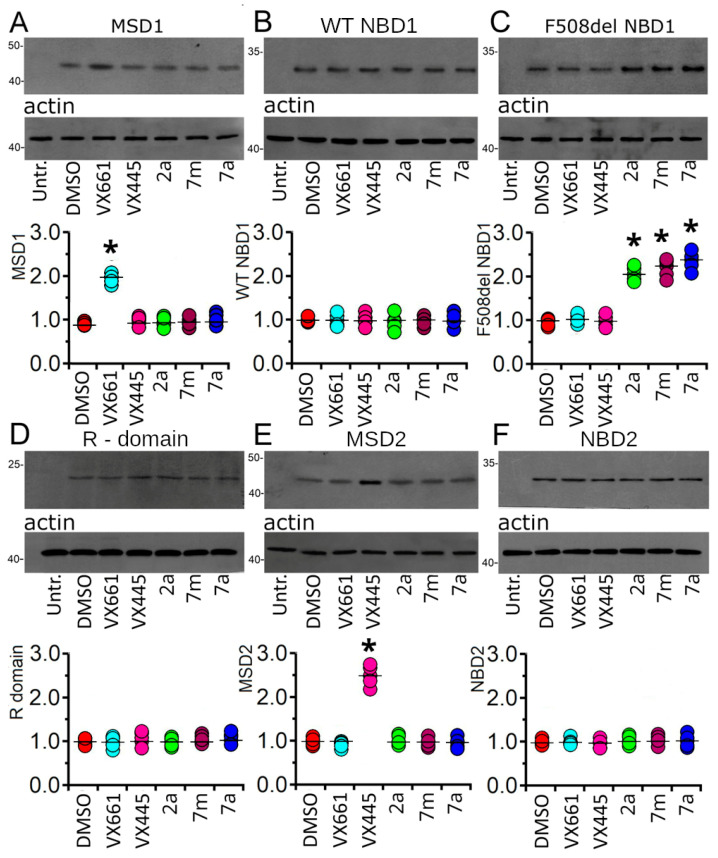
Effect of **VX661**, **VX445**, **2a**, **7m**, **7a** on CFTR single domain expression. Immunoblots of MSD1, (**A**) WT (**B**) and del508F NBD1, (**C**) R domain, (**D**) MSD2, (**E**) and NBD2 (**F**) CFTR domains in untransfected (untr) and transiently transfected HEK-t cells treated with the compounds under analysis are shown in the upper panels of the figure. The expression of actin, used as a housekeeping protein, is shown in the lower panels. The molecular weight of the proteins of the molecular weight marker run in the SDS-PAGE is given to the left of each blot. The graphs at the bottom of each panel show the normalised expression level of each domain. Black horizontal lines represent the mean ± standard error of the mean (SEM), while coloured circles represent single measurements (n ≥ 4). Dunnett’s post hoc multiple comparison test was used to compare data. Asterisks indicate statistical significance compared to control, DMSO-treated samples: * *p* < 0.05.

Western blot analysis of whole cell lysates from HEK-t cells transiently transfected with MSD1, WT and F508del NBD1, R domain, MSD2, and NBD2 was used to verify whether treatment with the compounds under investigation could determine an increase in the expression of a single domain of the CFTR protein. Retrieved immunoblots are shown in the upper panels of Figure 8A–F.

Antibodies raised against CFTR single domains revealed MSD1 as a polypeptide with an apparent molecular weight of ~45 kDa, whereas WT and F508del NBD1 were detected as electrophoretic bands of ~32 kDa. The R domain resulted in a protein with an apparent molecular weight of ~22 kDa. MSD2 and NBD2 were detected as bands of ~47 and ~30 kDa, respectively. Controls in untransfected HEK-t cells showed that the primary antibodies used in this work, whose characteristics are summarised in Table S5, did not detect any CFTR single domain on the blots.

As expected [38,48,49,50,51], treatment with **VX661** increased the expression of MSD1. Indeed, in HEK-t cells treated with **VX661**, the expression of this domain was 2.19 times higher than in control DMSO-treated cells. Neither **VX445** nor any of the newly synthetised hybrids altered the MSD1 expression (Figure 8A). None of the tested compounds modified the expression level, WT NBD1(Figure 8B). Similarly, **VX661** and **VX445** had no significant effect on the expression level of F508del NBD1. On the contrary, **2a**, **7m**, and **7a** induced a significant increase in the expression level of F508del NBD1. In particular, derivative **7a** was the most effective in increasing the expression of this mutant domain, determining an increase in its expression of 2.32 times with respect to that of control F508del NBD1DMSO-treated samples (Figure 8C). No significant increase in the expression level of the R domain polypeptide was observed after treatment with the compounds under investigation (Figure 8D). Treatment with the corrector, **VX661**, or with **2a**, **7m**, and **7a** did not change the expression of MSD2. On the contrary, as observed elsewhere [40,51], VX445 increased the expression of this domain to 2.51 times that observed in untreated control HEK-t cells (Figure 8E). Treatment with **VX661** or **VX445**, or with each of the three hybrids, did not modify the expression level of NBD2 (Figure 8F). The expression levels of the heterologously transfected CFTR single domains, together with the results of the statistical analyses, are shown in Supplementary Table S6.

#### 2.2.4. Effect of Tested Compounds on the Stability of F508del NBD1

**Figure 9:**
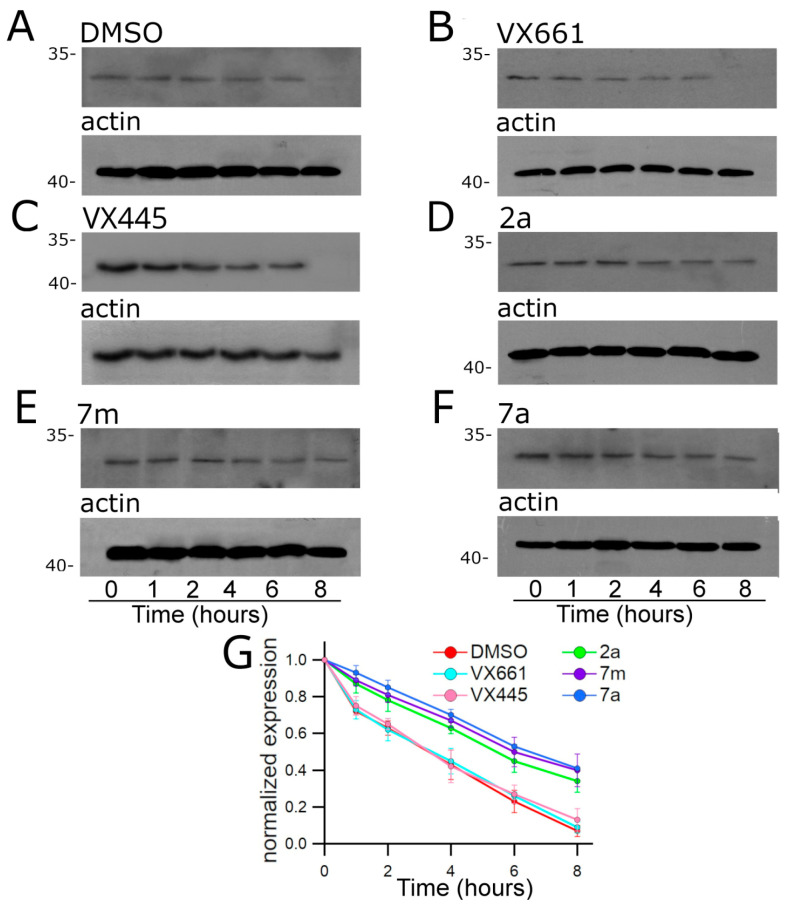
Stability of F508del NBD1, as assessed by the cycloheximide chase assay. The upper panels show the immunoblots of F508del NBD1 in HEK-t cell lysates treated with DMSO, (**A**) **VX661**, (**B**) **VX445**, (**C**) **2a**, (**D**) **7m**, (**E**) and **7a**, (**F**) subjected to protein synthesis inhibition by incubation with cycloheximide. Six different time points were analysed, as indicated at the bottom of the figure. The lower panels show the expression of actin as a housekeeping protein. The molecular weight of the proteins of the molecular weight marker run in the SDS-PAGE is indicated on the left of each blot. (**G**) Expression of F508del NBD1 protein at each time point. Data are expressed as mean ± SEM of at least 4 independent experiments. The symbols corresponding to the compounds assayed are indicated in the figure legend. The expression level of F508del NBD1 was normalised to the level of actin protein detected in the same samples and expressed relative to time 0.

Since **2a**, **7m**, and **7a** seemed to mainly affect the expression of F508del NBD1, we investigated whether these newly synthesised hybrids could have an effect on the stability of this polypeptide. For comparison, the effect of the treatment with the correctors, **VX661** and **VX445**, on the half-life of the F508del NBD1 polypeptide was also assayed (Figure 9).

Cycloheximide chase experiments showed that the expression of F508del NBD1 in control DMSO untreated samples decreased to 63% after 2 h, and to 43% and 23% after 4 h and 6 h from the beginning of the treatment with cycloheximide, respectively (Figure 9A). The time course decay of the expression level of F508del NBD1 in transfected HEK-t cell lysates treated with **VX661** and **VX445** was similar to that observed in control DMSO untreated samples. Indeed, in **VX661** and **VX445** treated samples, the expression level of F508del NBD1 was 62% and 65% after 2 h, from the blockade of protein synthesis with cycloheximide, and decreased to 45% and 26% in **VX661** treated samples, and to 42% and 27% of the initial one after 4 and 6 h, respectively (Figure 9B,C). In contrast, **2a**, **7m**, and **7a** determined a significant increase in F508del protein stability. Indeed, in **2a**, **7m**, and **7a** treated samples, the expression level of defective CFTR protein resulted in 78%, 81%, and 85% of the initial level at 2 h, 63%, 67%, and 70% at 4 h and 45%, 50%, and 53% at 6 h from the beginning of the treatment with cycloheximide, respectively (Figure 9D–F). Table S7 and Figure 9G show, for each time interval depicted in Figure 9, the values of F508del NBD1 expression, normalised to the intensity of actin, used as a housekeeping protein, and to the expression of F508del NBD1 at the beginning of the treatment with cycloheximide. For each condition, the statistical significance is also shown, as determined by Dunnett’s multiple comparison test (all groups versus control).

#### 2.2.5. Assessing the Impact of Corrector Combinations on Full-Length F508del CFTR Function

**Figure 10:**
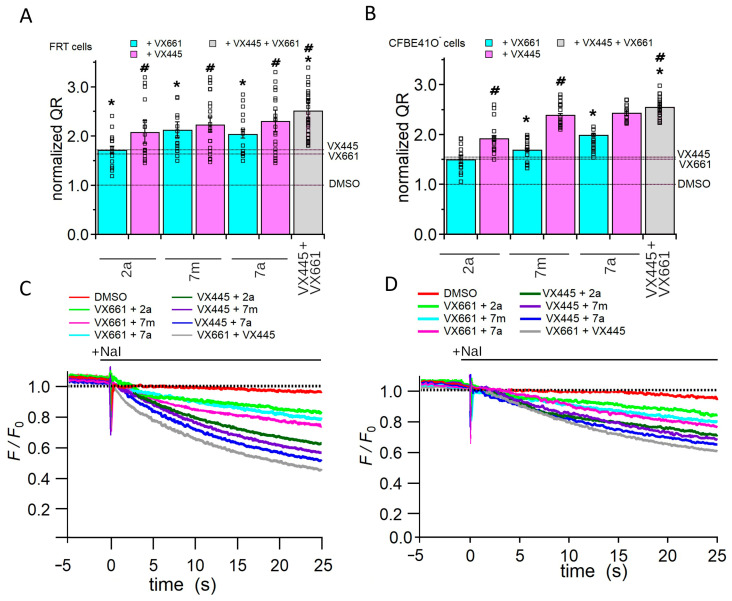
Evaluation of double combinations of the correctors herein explored on F508delCFTR channel function. (**A**) Quenching rate (QR) of the yellow fluorescent protein (YFP) elicited by the iodide influx in FRT cells permanently co-transfected with the YFP and the F508del CFTR proteins. (**B**) Time course of the fluorescence decay in FRT (**C**) and CFBE41O^−^ (**D**) cells. The fluorescence is normalised to the initial value obtained after the addition of iodide. Cells were treated with double combinations of the modulators evaluated in this work. In (**A**,**B**), the bars represent the mean ± standard error of the mean (SEM), while the black squares each single measurement. The dashed lines stand for the QR elicited in the cell preparations incubated with DMSO, **VX661**, or **VX445**, respectively. For each condition, each measurement (n) was repeated at least 10 times. Asterisks and hashtags (#) indicate a significant difference (* *p* < 0.05) with respect to **VX661** and **VX445** treated samples, respectively.

The YFP-based functional assay was used to investigate the effect of combinations of the compounds under investigation on F508del CFTR channel activity. FRT and CFBE41O^−^ cells stably expressing the F508del and YFP proteins were used for the experiments. As previously reported, incubation with forskolin and **VX770** was used to stimulate the maximal F508del channel activity (Figure 10A–D).

In particular, in both FRT and CFBE41O^−^ cells, the combination of **VX661** + **VX445** induced a greater increase the F508del CFTR iodide transport, which was significantly higher than that induced by **VX661** or **VX445** alone, respectively. In both cell lines, the effect of the combination **VX661** + **2a** on F508del CFTR channel activity was similar to that induced by **VX661** alone. On the contrary, the F508del CFTR-mediated I^−^ transport induced by the double combinations **VX661** + **7m**, and **VX661** + **7a** was significantly higher than that produced by **VX661** alone. In both cell lines, the combination **VX445** + **2a** also did not modify the QR with respect to **VX445**, whereas the hybrids, **7m** and **7a**, produced a significant additive/synergistic effect when combined with **VX445** (Figure 10A–D).

## 3. Discussion

In an attempt to answer the CF research challenge of finding effective therapy for people with CF who do not respond to current modulator treatment, we applied a multidisciplinary approach, including computational studies and biological assays of the in-house correctors, **2a**, **7a**, and **7m**. The final aim of the study was to elucidate the mechanism of action and evaluate the potential to work in combination of the aforementioned newly synthesised CFTR modulators, developed by merging the core of AAT with the benzodioxole carboxamide moiety that characterises the corrector, VX809 [35,36,37].

The first derivative we selected for the investigation was **2a**. This VX809 hybrid is characterised by a benzoyl group at the thiazole position 5, and shows good potency (EC_50_ = 0.087 µM) and promising efficacy as a CFTR corrector [36]. It was chosen as a prototype for the synthesis of other analogues, such as **7m** and **7a**, which carry structural modifications at the benzoyl moiety and show comparable or higher potency than **2a** (EC_50_ = 0.070 µM and E_C50_ = 0.10 µM, respectively [39]). As a first step, we used a structure-based computational analysis to identify the F508del CFTR region that might be primarily affected by or directly interact with the three newly synthesised hybrids. For this purpose, we: (i) collected and compared the most recently available cryo-electron microscopy (cryo-EM) structures of full-length F508del CFTR in the presence of modulators **VX809**, **VX661**, **VX445**, and **VX770** (Table 1) [40], (ii) evaluated the most appropriate docking protocol to reproduce these experimental data, (iii) applied the selected docking strategy to the in silico evaluation of the putative mechanism of action of **2a**, **7a**, and **7m**. This type of approach allowed us not only to confirm the presence of two cavities on the CFTR surface, one at the MSD1/NBD1 interface, which we called pocket B, and the other on the MSD2 surface, called pocked A, as sites of interaction between the correctors **VX661**, **VX809**, or **VX445** and the CFTR protein, but also to identify in silico the binding mode of **2a**, **7a**, and **7m** to CFTR in the presence of correctors belonging to different classes of modulators. In particular, we identified pocket B, as the most likely binding site of the three hybrids with their target protein, as shown by the reference correctors VX809 and VX661. There, the tested compounds resulted in π-π stacking and cation-π contacts with residues Trp361 and Arg74, or with Trp361 and Phe78 (Figure 5).

Since low toxicity is one of the criteria that a drug candidate must fulfill in order to proceed in the drug discovery process, before carrying out a series of biochemical experiments aimed at identifying the domains mainly involved in the rescue of the expression of the F508del CFTR protein promoted by the compounds under investigation, we carried out an evaluation of their toxicity. In all the cell lines used in this study, the trypan blue exclusion test showed relatively low toxicity values, not only for the correctors VX661 and VX445, but also for the newly synthesised hybrid compounds **2a**, **7m**, and **7a**. The retrieved results allowed us to select 3 µM for the correctors **VX661** and **VX445**, and 2 μM for the hybrids **2a**, **7m**, and **7a** as the concentrations to be used for further analysis.

Then, we analysed the effect of **2a**, **7m**, and **7a** on the expression of full-length WT and F508del CFTR, and on different domains of the CFTR protein, transfected individually in HEK-t cells, a heterologous expression system widely used because of its higher transfectability. Since the CFTR domains, whose expression is most affected by **VX445** and **VX661** are known [38,40,48,49,50,51], we used these correctors as reference compounds to compare results. Our analysis showed that in full-length WT CFTR lysates, neither the total (B + C bands) nor the mature (C/(C + B) band ratio) expression level of WT CFTR protein was increased after treatment with the correctors **VX661** and **VX445**, or each of the three hybrids under study (Figure 9A). On the contrary, all compounds demonstrated the ability to increase both total expression and the maturation of F508del CFTR (Figure 9B). In particular, in line with the results of previous studies [51], the next-generation corrector, VX445, exerted the strongest effect among the correctors tested, while 7a resulted in the most efficient compound among the hybrids (Figure 9B). Intriguingly, the different effects of the compounds under study on the expression levels of WT and F508del CFTR proteins lead us to hypothesise that these molecules may act selectively on those regions of the F508del CFTR, whose folding is different from that of the WT [52,53,54].

Alternatively, modulators could prevent the degradation of the mutant CFTR isoform by inhibiting a component of ERAD, the cellular machinery responsible for defective protein degradation, or by inducing the ER quality control complex to recognise the mutant CFTR as a protein prone to leave the ER compartment.

Another interesting finding of the present work concerns the analysis of the capability of the three hybrids to affect the F508del CFTR-mediated iodide transport in F508del-CFTR. Experiments were performed in FRT (Figure 10A,C) and CFBE41O^−^ (Figure 10B,D), cells stably expressing the YFP and the F508del CFTR. In both cell types, the compounds promoted F508del CFTR-mediated iodide transport activity in the following order: **VX445** > **VX661** > **7a** > **7m**> **2a**. The functional results obtained with the YFP assay correlate with the ability of the compounds to increase the expression of the F508del CFTR protein, and are consistent with the results obtained by our group and others for VX445, VX661, and other correctors [38,50,51].

Expression constructs encoding the MSD1 (M1, residues 1–388), NBD1 (N1, residues 348–633), WT and F508del NBD1, R (residues 645–834), MSD2 (M2, residues 837–1218), and NBD2 (N2, residues 1210–1480) CFTR domains were generated to identify the region of the mutant CFTR that is more susceptible to the action of the three hybrids under investigation [51].

Confirming previously reported results [38,50,51], the correctors **VX661** and **VX445**, which were used as reference compounds, increased the expression of MSD1 and MSD2, respectively, while they demonstrated an almost negligible effect on the expression levels of the other CFTR domains. Since the structure of the three hybrids is characterised by the benzodioxole carboxamide moiety that distinguishes **VX809** and its derivative, **VX661**, it was reasonable to assume that they would bind to MSD1, as their congeners, **VX809** and **VX661**, do. However, they do not induce any increase in the expression of this domain (Figure 8A). On the other hand, the presence of the aminoarylthiazole group in their scaffold would suggest that they could also bind to NBD2, as observed with the correctors, **FCG** and **Corr4a**, which share the same thiazolyl core. Surprisingly, **2a**, **7m**, and **7a** had no effect on the expression of NBD2(Figure 8F). They had no effect on the expression of the R domain (Figure 8D) and did not affect the expression of WT NBD1(Figure 8B). However, they were able to significantly increase the expression of the mutant, F508del NBD1 polypeptide (Figure 8C). Although we currently have no data to explain this impressive result, we can speculate that, similar to the full-length F508del CFTR, the misfolding of the F508del NBD1 determines the exposure of those residues that are specifically involved in the interaction between the mutant NBD1 domain and each of the three hybrids.

To definitively confirm that F508del NBD1 is the region mainly affected by the actions of **2a**, **7m**, and **7a**, we examined the effect of the three hybrids on the stability of this polypeptide. We hypothesised that if **2a**, **7m**, and **7a** act by stabilising the region whose expression they increase, then this region would be expected to have a slower turnover rate after F508del NBD1 transiently transfected HEK-t cells were treated with cycloheximide to inhibit protein synthesis. Therefore, HEK-t cells were transfected with plasmids encoding F508del NBD1 and then incubated with **VX661**, **VX445**, **2a**, **7m**, **7a**, or DMSO as a control vehicle. The next day, protein synthesis was inhibited with cycloheximide. Whole-cell extracts were collected at different time points and analysed by SDS-PAGE and Western blot analysis. The decrease in F508del NBD1 expression was 43% and 23% in control DMSO treated samples after 4 and 6 h, respectively (Figure 7A). Consistent with previous results [38,50,51], both **VX661** and **VX445** did not prolong the half-life of F508del NBD1 (Figure 7B,C). On the contrary, the newly synthesised hybrids extended the half-life of the F508del NBD1 domain. Indeed, the expression level of F508del NBD1 after 2, 4, and 6 h from the beginning of the treatment with cycloheximide was significantly higher in **2a**, **7m**, and **7a** treated samples than in control DMSO treated samples (Figure 7D–G).

Since it is widely acknowledged that the allosteric effect of two or more modulators targeting distinct defects of the mutant CFTR protein leads to a significant increase in its functional expression [7,26,27], we tested the ability of combinations of the newly synthesised hybrids with correctors already in clinical use to increase the activity of the F508del CFTR channel. In particular, we used **VX661**, a class I corrector thought to support the formation of the NBD1/MSD1 and NBD1/MSD2 interfaces by binding directly to MSD1 [40,44], and **VX445**, which acts by increasing the expression and stability of MSD2 [38,40,48,49,50,51].

Our analysis showed that the three hybrids exert their effect by binding to and enhancing the expression of mutant F508del NBD1. Consequently, the rescue of more than one of the different defects of the F508del protein produced by the combined activity of each of the three hybrids with **VX661** or **VX445** should produce an increase in mutant CFTR functionality higher than that of each compound administered alone. Interestingly, when we examined the efficacy of the three hybrids in combination with **VX661** to increase F508del CFTR mediated iodine transport, we observed that in FRT and CFBE41O^−^ cells, two of the three hybrids, **7m** and **7a**, showed additive/synergistic effects with **VX661** (Figure 8A–C). The combination of **7m** and **7a** with **VX445** produced an even more pronounced increase in F508del CFTR channel activity (Figure 8B–D). In contrast, none of the combinations with compound **2a** produced an effect greater than that produced by **2a**, **VX661**, or **VX445**, administered singly. This unexpected result could be due to the lower potency of this prototype compared to the next-generation hybrids, **7m** and **7a**, or to the allosteric inhibition of the binding to the target site on the F508del NBD1 domain exerted by the correctors, **VX661** and **VX445**. It is worth noting that the YFP assay indicated that in CFBE41O^−^, the cell line that almost recapitulates the characteristics of human airway epithelial cells, the improvement in F508del channel function obtained by the combination of hybrid **7a** with **VX445** is comparable to that obtained by **VX445** in combination with the type I corrector **VX661** (Figure 8A–D).

## 4. Materials and Methods

### 4.1. Computational Studies

All the in silico explored compounds were manually built thanks to the MOE software 2019.01 version (Builder tool) [55], and then parametrised, referring to the AM1 method (partial charge calculation), and by energy minimisation via the Energy Minimise tool implemented in MOE. The MMFF94x forcefield and RMS (root mean square) gradient equal to 0.0001 were applied. The RMS gradient represents the norm of the gradient times the square root of the number of (unfixed) atoms. This kind of calculation allowed us to obtain a single low-energy conformation for each ligand [55].

All the exploited experimental data of the CFTR protein in the presence of different ligands were downloaded from the Protein Data Bank [56,57] and managed in silico via QuickPrep module implemented in MOE software, prior to the following computational studies.

All the molecular docking calculations have been performed, applying the DOCK tool implemented in MOE based on the template similarity methodology, including all those amino acids placed at 4.5 Å distance from the PDB ligand in the experimental conformation. Details of the docking procedure have been previously described by us [58,59].

Calculation of the enthalpy-based Affinity dG scoring function (S score) allowed us to rank fifty poses prior to the final ten ones for each corrector, after pose refinement.

### 4.2. Chemicals

The molecules, **2a**, **7a**, and **7m**, were synthetised, as previously published [37,38]. The known CFTR modulators, Tezacaftor, VX661, Elexacaftor, VX445 and Ivacaftor, and VX770 were purchased from Selleck Chemicals (Munich, Germany). If not explicitly indicated in the text, all other chemicals and culture media components were provided by Merck (Milan, Italy).

### 4.3. Biochemical Assays

#### 4.3.1. Cell Culture and Compound Toxicity Evaluation

Human highly transfectable embryonic kidney 293 (HEK293-t) cells were purchased from the Interlab Cell Line Collection (Genoa, Italy) and grown in Dulbecco’s modified Eagle’s medium (DMEM), supplemented with 2 mM L-glutamine, 1% PenStrep (100 U/mL), and 20% FBS, at 37 °C and 5% CO_2_. Fisher rat thyroid (FRT) and human bronchial epithelial F508del CFBE41O^−^ (CFBE41O^−^) cells stably co-transfected with a halide-sensitive yellow fluorescent protein (YFP-H148Q/I152L [60,61]), and F508del-CFTR were cultured in Coon’s modified and MEM media, respectively. In both cases, media were supplemented with 10% FBS, 2 mM L-Glutamine, 1% PenStrep (100 U/mL), 1 mg/mL geneticin (G418), and 0.6 mg/mL zeocin as selection agents. Cells were grown under standard CO_2_ and temperature conditions. Confluence of cell was avoided to prevent loss of differentiation.

Toxicity of **2a**, **7m**, **7a**, **VX661** and **VX445** in HEK-t, FRT, and CFBE41O^−^ cells was evaluated by the Trypan blue exclusion staining method [62]. Cells were seeded into 48-well plates at a concentration of 0.3 × 10^5^ cells per well. The day after seeding, cells were exposed to the compounds for 24 h at the following concentrations: 10, 5, 3, 2, 1, 0.5, 1, 0.5, 0.25, 0.125, and 0 (vehicle, DMSO) μM. After exposition, cells were trypsinised and harvested for toxicity evaluation. To avoid an underestimation of the number of dead cells, the cells that were detached from the plates during exposure to the compounds were collected and considered. For each condition tested, at least four technical replicates were evaluated. In each well, at least 100 cells were considered. The concentration resulting in the half maximum toxicity, TD_50_, was calculated by plotting the percentage of cell survival against the concentration of the analysed compound (C) and fitting the data with a sigmoidal function.

#### 4.3.2. Generation and Expression of CFTR Constructs

Plasmidic DNA encoding the entire CFTR molecule (residues 1–1480), MSD1 (residues 1–388M), NBD1 (residues 348–633), MSD2 (residues 837–1218), and NBD2 (residues 1210–1480) was digested between Hind III and XhoI, and encoded the R domain (residues 645–834) between the Hind III and EcoRI restriction sites, and inserted into the expression vector, pCDNA3 (Invitrogen, Paisley, UK) [38,50,51]. Site-directed mutagenesis (QuickChange kit, Stratagene, Santa Clara, CA, USA) was used to generate the deletion of the nucleotides encoding the phenylalanine at position 508 of the CFTR molecule. The correctness of the procedure was verified by sequencing (Biofab Research, Rome, Italy).

For transfection, 0.8 × 10^6^ HEK-t cells were plated onto 60 mm poly-L-lysine-coated culture dishes and grown to 65% confluence in a complete medium. Cells were transiently transfected using Lipofectamine 2000 (Invitrogen, Paisley, UK) with 4 μg of cDNA. The transfection medium was replaced after 6 h with a fresh complete medium (DMEM supplemented with 2 mM L-glutamine and without FBS) containing 2 μM **2a**, 2 μM **7m**, 2 μM **7a**, 3 µM **VX661**, 3 μM **VX445**, or vehicle DMSO (control). Cells were harvested after 24 h.

#### 4.3.3. Western Blot

Cells were grown to confluence on 60 mm diameter dishes and lysed in RIPA buffer (50 mM Tris-HCl, pH 8.0, 150 mM NaCl, 1% Triton X-100, 1% sodium deoxycholate, 0.1% SDS) containing a complete protease inhibitor cocktail (Sigma-Aldrich, Milan, Italy). Cell lysates were centrifuged at 12,000 rpm for 10 min at 4 °C. The protein concentration of the supernatant was calculated by the Bradford method using bovine serum albumin as a standard. Equal amounts of protein (30 μg) were subjected to SDS-PAGE and transferred to a PVDF membrane (Millipore, Billerica, MA, USA). Blots were incubated with primary antibodies raised against different domains of the CFTR protein, the characteristics of which are summarised in Supplementary Table S7. Secondary antibodies were goat anti-mouse or anti-rabbit horseradish peroxidase-conjugated antibodies (1:2000 dilution; Santa Cruz Biotechnologies, Dallas, TX, USA). Results were visualised by chemiluminescence using Amersham ECL PLUS detection reagents (GE Healthcare Europe GmbH, Milan, Italy), and images were captured using Amersham Hyperfilm ECL. Images were analysed using ImageJ software (National Institutes of Health). The intensity of the bands was analysed as a region of interest (ROI). For quantification, the intensity of the bands in each lane was normalised to the actin loading control. Data are expressed as mean ± SEM of at least four independent experiments.

#### 4.3.4. Cycloheximide Chase Assay

The stability of NBD1-F508del was evaluated according to a previously established procedure [38,50,51]. Briefly, HEK-t cells were transfected with the plasmid containing the cDNA encoding this construct and incubated for 24 h in the presence of 2 μM **2a**, 2 μM **7m**, 2 μM **7a**, 3 µM **VX661**, 3 μM **VX445**, or vehicle DMSO (control). Protein synthesis was then inhibited by the addition of 0.5 mg/mL cycloheximide. Cells were harvested at six different time points (after 0, 1, 2, 4, 6, and 8 h), and samples of whole-cell SDS extracts were subjected to immunoblot analysis.

#### 4.3.5. YFP Functional Assay

One day after seeding on black-walled, clear-bottomed 96-well microplates at a density of 30,000 cells per well, FRT or CFBE41O cells stably co-transfected with a halide-sensitive yellow fluorescent protein (YFP-H148Q/I152L [60,61]) and F508del CFTR were incubated for 18 h with DMSO (control vehicle), 2 μM **2a**, 2 μM **7m**, 2 μM **7a**, 3 µM **VX661**, 3 μM **VX445**, or double or triple combinations of the tested correctors. At the time of the assay, cells were washed twice with phosphate-buffered saline (PBS) containing (in mM) NaCl 136, KNO_3_ 4.5, Ca(NO_3_)_2_ 1.2, MgSO_4_ 0.2, glucose 5, HEPES 20 (pH 7.4). Cells were then incubated with 60 µL PBS plus forskolin (20 µM) and **VX-770** (1 µM) for 25 min to maximally stimulate F508del-CFTR. The cells were then transferred to a microplate reader (Tristar2 S, Berthold Technologies, Bad Wildbad, Germany) equipped with 485 nm excitation and 535 nm emission filters.

The assay consisted of a fluorescence reading every 0.2 s, 5 s before and 25 s after injection of 100 μL of an extracellular solution containing 136 mM NaI (PBS with Cl^−^ replaced by I^−^; final I^−^ concentration, 85 mM). Iodide influx was detected as fluorescence quenching as the I^−^ anion bound to intracellular YFP. After background subtraction and normalisation to the average fluorescence before NaI addition, the initial fluorescence decay rate (QR) was derived by fitting the signal with an exponential function.

#### 4.3.6. Data analysis

Igor Pro software (version 9.0.2.4, Wavemetrics, Lake Oswego, OR, USA) was used for data analysis. Results are expressed as mean ± SEM (standard error of the mean). One-way analysis of variance (ANOVA) followed by Dunnett’s post hoc multiple comparison test was used to compare datasets. Significance was accepted at a probability of *p* < 0.05.

## 5. Conclusions

To summarise, three main outcomes have been achieved in this work. First, we have established a computational protocol that could be usefully applied to guide the design of optimised lead compounds to obtain more potent CFTR modulators. Secondly, although we have not provided direct evidence of protein-ligand interaction, our results indicate that F508del NBD1 is their target domain. Thirdly, the results obtained in this study provide further evidence that the use of personalised combinations of CFTR modulators, able to rescue the specific molecular and cellular defects caused by CF-causing mutations, is the strategy of choice to finally overcome CF disease.

## Figures and Tables

**Table 1:** Cryo-electron microscopy (cryo-EM) data regarding the human CFTR protein, as released from 2017. The experimental data herein exploited for the following structure-based studies are highlighted in light cyan and are referred to the human F508del CFTR. The other PDB codes include WT CFTR protein structures.

PDB Code	Feature	Resolution (Å)	Release	Atom Count	Reference
8EIQ	ELEXACAFTOR, TEZACAFTOR. IVACAFTOR	3.00	2022	9578	[40]
8EIO	ELEXACAFTOR, LUMACAFTOR	2.80	2022	9550	[40]
8EIG	ELEXACAFTOR	3.60	2022	9249	[40]
7SVR	LUMACAFTOR NO COFACTOR	3.90	2022	7841	[41]
7SVD	COFACTOR ATP (ADENOSINE-5′-TRIPHOSPHATE)	2.70	2022	9635	[41]
7SV7	TEZACAFTOR NO COFACTOR	3.80	2022	9673	[41]
6O2P	IVACAFTOR COFACTOR ATP (ADENOSINE-5′-TRIPHOSPHATE)	3.30	2019	9767	[42]
6O1V	GLPG1837 COFACTOR ATP (ADENOSINE-5′-TRIPHOSPHATE)	3.20	2019	9711	[42]
6MSM	COFACTOR ATP (ADENOSINE-5′-TRIPHOSPHATE)	3.20	2018	9703	[43]
5UAK	NO COFACTOR	3.87	2017	9232	[5]

**Table 2:** The best ranked docking poses of the hybrids **2a**, **7a**, and **7m** and of the CF drugs **VX809**, **VX661**, and **VX445** are listed via MOE Dock. The number of different conformer clusters (CLs) obtained during molecular docking calculations is reported (CLN) in tandem with the corresponding best S-score values (affinity dG; Kcal/mol) of CL1. The population of CL1 defined as the one containing the highest score pose is also reported.

	8EIG-VX445 Binding Site (Pocket A)			8EIO-VX809 Binding Site (Pocket B)			8EIQ-VX661 Binding Site (Pocket B)		
Compounds	CL1 S Value	CL1 Population	CLN	CL1 S Value	CL1 Population	CLN	CL1 S Value	CL1 Population	CLN
**VX-445**	−6.5860	8	2	−7.3970	8	2	−7.9867	3	5
**VX-809**	−5.4008	5	3	−8.7534	5	2	−9.0052	6	2
**VX-661**	−5.8536	8	3	−9.7202	9	2	−9.9823	8	2
**2a**	−4.4228	3	5	−7.8760	7	3	−8.3534	6	2
**7a**	−4.6130	4	4	−8.5604	6	2	−8.5864	4	4
**7m**	−5.5739	5	4	−10.3685	6	3	−8.8892	6	4

**Table 3:** Effect of the compounds under investigation on cell viability. The viability of HEK-t, FRT, and CFBE41O^−^ cells was assessed by the Trypan blue exclusion test after 24 h incubation with **VX661**, **VX445**, **2a**, **7m**, and **7a** at the following concentrations: 10, 5, 3, 2, 1, 0.5, 1, 0.5, 0.25, 0.125, and 0 (vehicle, DMSO) μM. TD_50_ is the concentration required to achieve half maximum toxicity, whereas TD_max_ is the maximum toxicity. Data are presented as means ± SEM of at least four independent experiments.

	HEK-t		FRT		CFBE41O^−^	
Compound	TD_50_ (µM)	TD_Max_ (µM)	TD_50_ (µM)	TD_Max_ (µM)	TD_50_ (µM)	TD_Max_ (µM)
**VX661**	27.15 ± 17.88	56.42 ± 0.30	11.09 ± 3.22	23.59 ± 0.08	7.51 ± 2.22	15.62 ± 0.06
**VX445**	9.60 ± 2.25	20.75 ± 0.04	4.29 ± 0.32	10.79 ± 0.01	11.75 ± 3.42	23.60 ± 0.07
**2a**	2.44 ± 1.03	5.78 ± 0.04	9.24 ± 2.25	20.56 ±0.06	10.11 ± 1.80	14.44 ± 0.06
**7m**	1. 40 ± 0.20	5.86 ± 0.01	5.76 ± 0.89	13.68 ± 0.03	9.78 ± 3.35	29.51 ± 0.10
**7a**	1.59 ± 0.36	5.82 ± 0.01	6.13 ± 2.75	14.70 ± 0.07	11.97 ± 2.87	25.49 ± 0.08

## Data Availability

The data presented in this study are available upon request from the corresponding author.

## Supplementary Materials

The following supporting information can be downloaded at: https://www.mdpi.com/article/10.3390/ph16121702/s1, Figure S1: Comparison of the ligplot schemes concerning the 8EIG, 8EIO, and 8EIQ experimental data; Figure S2. Docking pose of the corrector, **VX809**, at the 8EIO PDB code; Figure S3: Docking pose of corrector, **VX661**, at the PDB code, 8EIQ; Figure S4: Experimental pose of **VX809** at the 7SVD PDB code; Figure S5: Experimental pose of **VX661** at the 7SV7 PDB code. Table S1: Ten top scored docking positioning at the PDB code 8EIG of the studied CFTR modulators; Table S2: Ten top scored docking positioning at the PDB code, 8EIO of the studied CFTR modulators; Table S3: Ten top scored docking positioning at the PDB code, 8EIQ of the studied CFTR modulators; Table S4: Evaluation of WT and F508del CFTR protein expression in HEK-t cells whole-cell lysates after treatment with hybrids; Table S5: Evaluation of CFTR single-domain expression in HEK-t cells whole-cell lysates after treatment with the studied CFTR modulators; Table S6: Evaluation of F508del NBD1 protein expression in HEK-t cells whole-cell lysates after treatment with the compounds under investigation; Table S7: Primary antibodies used to detect whole length WT and F508del CFTR, and CFTR single domains used in this work.
