# Supplementary material for: In Silico and In Vitro Evaluation of the Mechanism of Action of Three VX809-Based Hybrid Derivatives as Correctors of the F508del CFTR Protein
Source: Pharmaceuticals (Basel). 2023 Dec 8;16(12):1702. doi: 10.3390/ph16121702 (PMC10748060; doi:10.3390/ph16121702)
Supplement: Supplementary file 1 [file pharmaceuticals-16-01702-s001.zip › pharmaceuticals-2741224-supplementary.pdf]

SUPPORTING INFORMATION

In Silico and In Vitro Evaluation of the Mechanism of Action of Three VX809-Based Hybrid Derivatives as Correctors of the F508del CFTR Protein

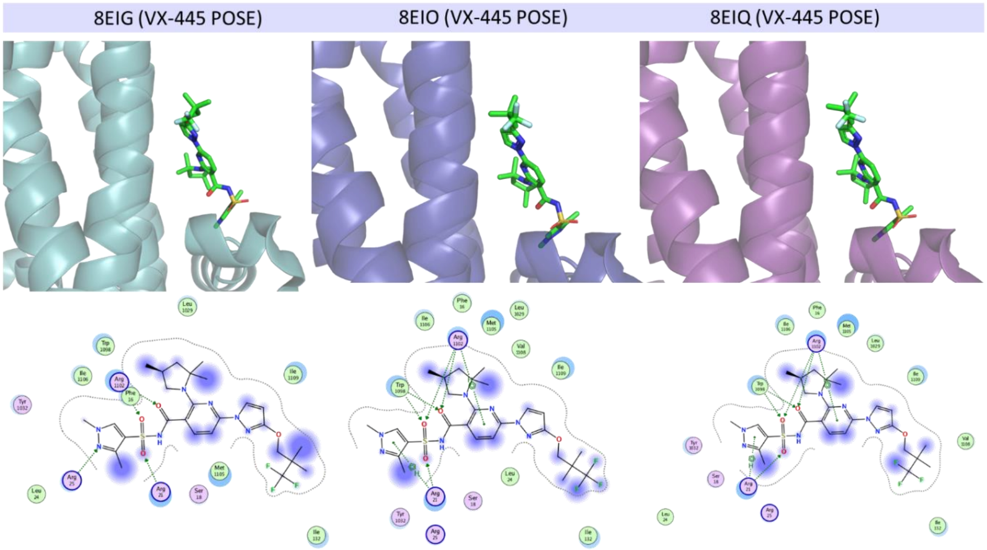

Figure S1. Comparison of the ligplot schemes concerning the 8EIG, 8EIO and 8EIQ experimental data. Polar and hydrophobic residues are colored in pink and green, respectively. The ligand portion to be projected outside the protein surface are highlighted in blue.

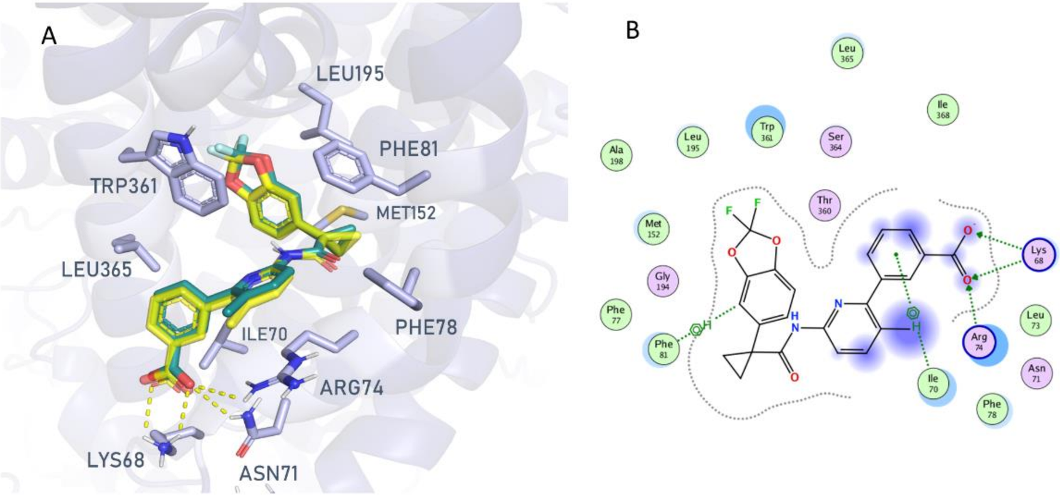

Figure S2. Docking pose of the corrector **VX809** (C atom; green) at the 8EIO PDB code, with respect to the experimental positioning (C atom; yellow) (A) Ligplot as a schematic representation of the main protein-ligand contacts is reported (B). Polar and hydrophobic residues are colored in pink and green, respectively. The ligand portion to be projected outside the protein surface are highlighted in blue.

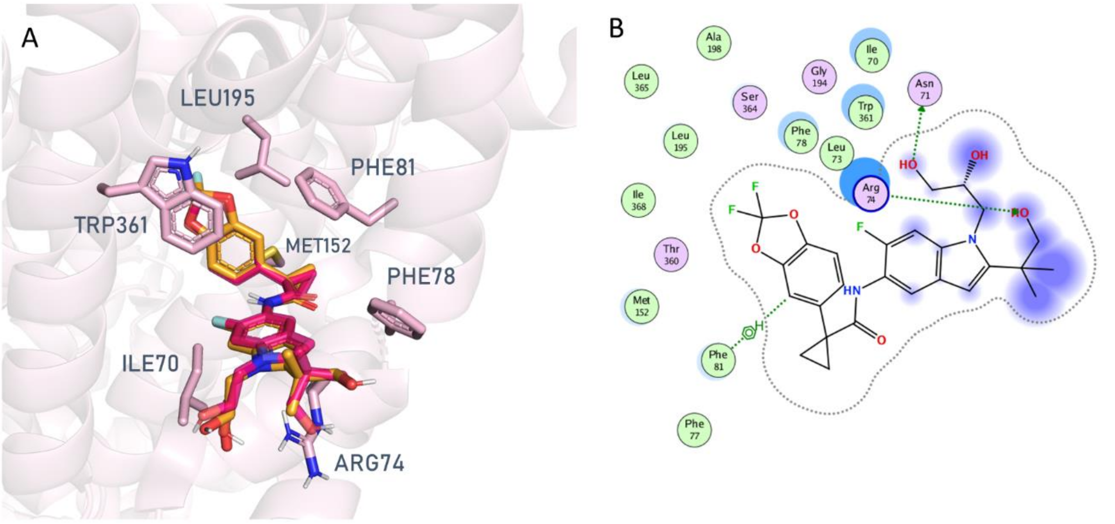

Figure S3. Docking pose of corrector **VX661** (C atom; orange) at the PDB code 8EIQ, with respect to the experimental positioning (C atom; magenta) (A) Ligplot as a schematic representation of the main protein-ligand contacts is reported (B). Polar and hydrophobic residues are coloured in pink and green, respectively. The ligand portion to be projected outside the protein surface is highlighted in blue.

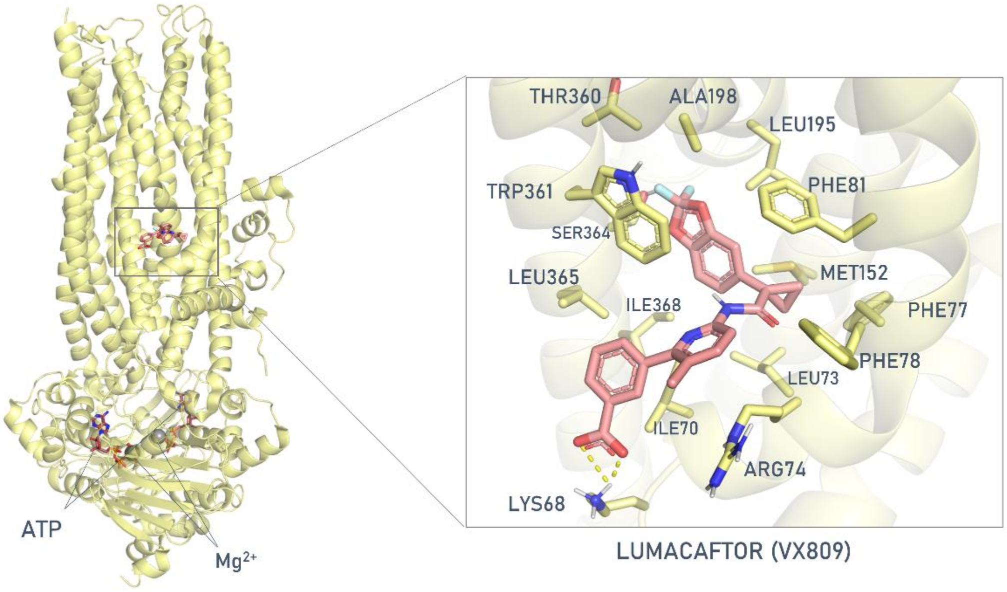

Figure S4. Experimental pose of **VX809** (C atom; pink) at the 7SVD PDB code. The whole protein is depicted (left side) being the corresponding protein-ligand binding mode also shown (right side).

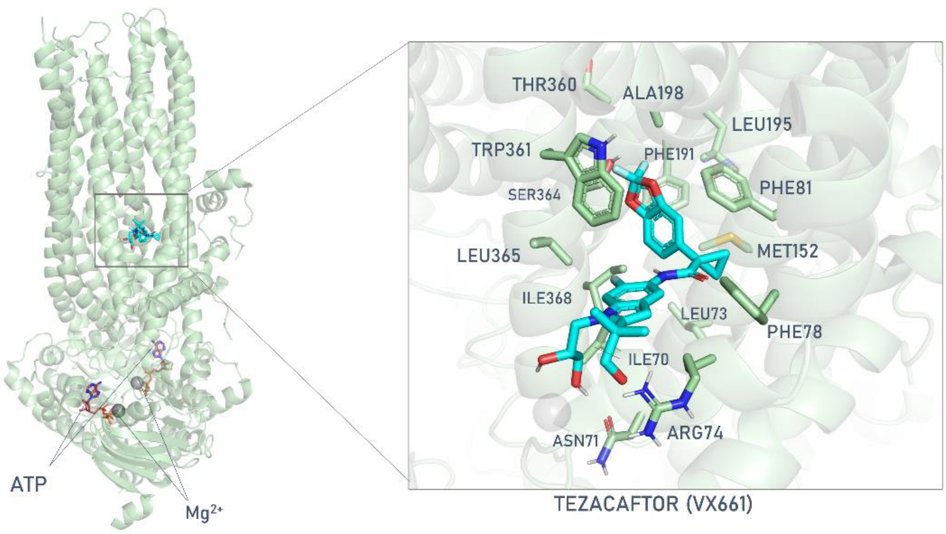

Figure S5. Experimental pose of **VX661** (C atom; cyan) at the 7SV7 PDB code. The whole protein is depicted (left side) being the corresponding protein-ligand binding mode also shown (right side).

Table S1. Ten top scored docking positioning at the PDB code 8EIG of the herein investigated hybrids **2a**, **7a**, **7m** and of the reference CFTR modulators **VX809**, **VX661** and **VX445**. The predicted ΔG value of each protein-ligand complex has been reported, as calculated in terms of final scoring function (S, as Kcal/mol).

Compound	S	E_conf	E_place	E_score1	E_refine	E_score2
**2a**	-4.4228	56.9634	-18.3158	-9.1392	-15.5229	-4.4228
**2a**	-4.4099	58.6776	-9.1591	-8.4493	-22.1654	-4.4099
**2a**	-4.2118	51.1288	-11.6429	-8.4974	-16.7278	-4.2118
**2a**	-4.1333	50.2729	-25.3931	-8.3503	-18.8067	-4.1333
**2a**	-4.1037	62.2578	-22.7305	-8.3799	-17.4027	-4.1037
**2a**	-3.7519	57.0222	-14.8068	-8.7094	-17.8910	-3.7519
**2a**	-3.7480	60.4186	-10.9114	-8.5670	-15.8403	-3.7480
**2a**	-3.6337	57.0071	-11.2084	-8.5839	-14.3311	-3.6337
**2a**	-3.6122	67.5482	-11.7842	-8.3229	-14.6793	-3.6122
**2a**	-3.5785	57.8832	-18.8391	-8.6674	-14.7912	-3.5785
**7a**	-4.6130	54.7758	-10.3368	-7.5923	-15.6945	-4.6130
**7a**	-4.3594	52.4895	-14.9376	-8.2332	-15.0947	-4.3594
**7a**	-4.2645	56.3198	-19.4090	-8.3653	-17.9217	-4.2645
**7a**	-4.2626	56.8435	-12.6205	-8.1601	-18.5669	-4.2626
**7a**	-4.2530	54.0131	-17.4000	-9.0553	-15.2420	-4.2530
**7a**	-4.2305	51.2142	-11.8824	-7.5602	-14.6812	-4.2305
**7a**	-4.1679	65.9758	-20.9108	-7.6579	-24.8061	-4.1679
**7a**	-4.0571	52.7103	-13.9638	-7.4941	-17.4643	-4.0571
**7a**	-4.0371	56.4814	-15.2284	-8.5400	-18.2004	-4.0371
**7a**	-3.8821	57.4821	-16.6414	-7.6769	-20.0048	-3.8821
**7m**	-5.5739	82.5771	-19.3154	-8.5506	-17.8229	-5.5739
**7m**	-5.5070	78.7220	-17.4841	-9.1314	-20.4701	-5.5070

**7m**	-5.3241	77.8900	-23.2073	-7.7716	-21.0670	-5.3241
**7m**	-5.2947	78.3826	-13.3029	-7.7725	-17.6208	-5.2947
**7m**	-5.1294	90.1531	-14.4151	-8.2315	-26.2099	-5.1294
**7m**	-5.0461	77.9617	-23.0444	-7.7261	-16.4003	-5.0461
**7m**	-4.9924	72.2097	-24.1816	-7.8483	-18.7974	-4.9924
**7m**	-4.9701	71.7269	-22.4046	-8.3210	-21.5056	-4.9701
**7m**	-4.9392	77.5923	-28.5912	-10.4936	-21.4183	-4.9392
**7m**	-4.7844	73.6395	-13.6819	-7.8326	-19.9741	-4.7844
**VX809**	-5.4008	-44.3477	-16.3145	-10.1315	-19.4386	-5.4008
**VX809**	-4.5365	-45.6606	-18.7366	-10.3997	-21.9572	-4.5365
**VX809**	-4.4755	-45.5444	-18.4278	-10.8512	-21.6917	-4.4755
**VX809**	-4.2128	-37.8443	-19.8072	-9.9893	-22.2434	-4.2128
**VX809**	-4.1660	-46.2295	-16.2937	-9.7170	-16.9632	-4.1660
**VX809**	-4.1471	-45.9700	-18.3068	-9.8728	-17.5237	-4.1471
**VX809**	-3.8710	-45.1056	-13.0989	-9.3610	-15.3866	-3.8710
**VX809**	-3.8554	-44.7563	-16.8875	-10.6660	-14.4862	-3.8554
**VX809**	-3.8307	-46.3563	-21.9392	-9.4455	-13.2429	-3.8307
**VX809**	-3.8199	-42.0960	-16.3085	-9.4833	-12.0893	-3.8199
**VX445**	-6.5860	24.2476	-37.9831	-9.5865	-26.7786	-6.5860
**VX445**	-6.5409	22.8789	-20.5021	-8.4063	-34.7520	-6.5409
**VX445**	-6.3788	24.4139	-31.8946	-8.1334	-27.9866	-6.3788
**VX445**	-6.1390	30.1084	-30.8127	-8.5172	-23.8528	-6.1390
**VX445**	-6.0727	20.3749	-19.7641	-8.7947	-31.7252	-6.0727
**VX445**	-5.6625	26.6032	-19.7742	-9.5048	-24.8288	-5.6625
**VX445**	-5.5202	24.7748	-17.7726	-8.8854	-29.8918	-5.5202
**VX445**	-5.1298	25.9926	-19.8374	-9.1308	-26.7523	-5.1298
**VX445**	-4.9616	32.9035	-30.3498	-9.1653	-24.7015	-4.9616
**VX445**	-4.9341	20.3617	-17.2918	-8.6630	-18.7333	-4.9341
**VX661**	-5.8536	89.1681	-25.2313	-9.5894	-19.1907	-5.8536
**VX661**	-4.8274	88.3569	-25.0704	-8.7452	-17.9080	-4.8274
**VX661**	-4.6785	88.5221	-16.6701	-9.9013	-17.8667	-4.6785
**VX661**	-4.6338	87.5115	-19.4357	-9.3553	-12.6473	-4.6338
**VX661**	-4.5808	90.1363	-16.8477	-8.8757	-18.8349	-4.5808
**VX661**	-4.5602	94.0658	-13.0931	-10.1650	-15.3349	-4.5602
**VX661**	-4.5362	93.9468	-29.7441	-9.6855	-14.4733	-4.5362
**VX661**	-4.3610	99.6229	-22.1399	-9.4511	-9.2065	-4.3610
**VX661**	-4.3421	88.8984	-14.4665	-9.0677	-13.8675	-4.3421
**VX661**	-4.3036	87.4641	-17.5855	-8.7127	-13.8601	-4.3036
**VX770**	-4.3525	67.6797	-16.9569	-8.5330	-16.8782	-4.3525
**VX770**	-4.3219	67.2216	-17.0488	-8.6016	-14.2117	-4.3219
**VX770**	-4.2726	79.7864	-20.7953	-8.9535	-13.2258	-4.2726
**VX770**	-4.1813	67.3965	-24.6563	-8.7471	-17.5856	-4.1813
**VX770**	-4.1799	69.7318	-21.6881	-8.5389	-18.2055	-4.1799
**VX770**	-4.1006	70.8510	-21.0288	-8.8552	-16.7869	-4.1006
**VX770**	-4.0835	69.3895	-23.4633	-8.9818	-15.5897	-4.0835
**VX770**	-4.0317	69.4344	-16.6557	-8.6268	-15.7996	-4.0317

**VX770**	-3.9886	70.6607	-19.9442	-9.0446	-16.0518	-3.9886
**VX770**	-3.5944	68.6958	-14.3132	-10.4784	-12.4640	-3.5944

Table S2. Ten top scored docking positioning at the PDB code 8EIO of the herein investigated hybrids **2a**, **7a**, **7m** and of the reference CFTR modulators **VX809**, **VX661** and **VX445**. The predicted ΔG value of each protein-ligand complex has been reported, as calculated in terms of final scoring function (S, as Kcal/mol).

Compound	S	E_conf	E_place	E_score1	E_refine	E_score2
**2a**	-7.8760	116.0195	-23.5390	-7.7545	-35.7797	-7.8760
**2a**	-7.6524	112.8703	-30.9584	-7.5365	-45.9355	-7.6524
**2a**	-7.4779	110.3908	-32.1541	-8.0422	-39.3087	-7.4779
**2a**	-7.2521	113.1609	-31.9264	-8.0629	-34.7592	-7.2521
**2a**	-7.0345	115.1808	-16.2659	-7.5219	-28.0563	-7.0345
**2a**	-6.8592	109.4064	-40.8402	-9.3119	-44.8716	-6.8592
**2a**	-6.8227	133.6937	-32.7090	-8.1424	-17.2157	-6.8227
**2a**	-6.7879	117.7266	-15.2858	-7.5962	-23.2209	-6.7879
**2a**	-6.7514	135.2815	-33.9475	-9.1055	-16.0762	-6.7514
**2a**	-6.6382	133.0922	-25.4548	-8.6742	-20.3167	-6.6382
**7a**	-8.5604	106.8645	-36.0003	-9.4109	-44.9491	-8.5604
**7a**	-8.3779	108.8403	-29.9209	-7.7165	-46.9429	-8.3779
**7a**	-8.1756	121.9859	-27.2932	-7.8331	-6.8057	-8.1756
**7a**	-8.1714	108.2967	-21.0676	-7.2808	-49.7397	-8.1714
**7a**	-8.0876	105.4895	-16.4576	-7.2870	-46.7500	-8.0876
**7a**	-8.0775	107.9158	-23.6461	-8.3844	-48.0181	-8.0775
**7a**	-8.0243	108.0966	-20.5120	-7.4902	-30.5772	-8.0243
**7a**	-7.9423	111.4310	-36.4321	-7.8171	-42.0793	-7.9423
**7a**	-7.6852	130.5894	-27.2734	-7.4069	-10.2409	-7.6852
**7a**	-7.3963	110.1238	-27.9648	-7.3559	-31.1471	-7.3963
**7m**	-10.3685	122.2545	-16.0101	-7.6162	-39.7925	-10.3685
**7m**	-10.2723	116.8313	-18.1172	-8.9993	-37.2291	-10.2723
**7m**	-9.7114	121.0419	-19.3945	-8.4804	-38.2188	-9.7114
**7m**	-9.5662	117.8600	-28.0876	-7.6774	-42.9933	-9.5662
**7m**	-9.3946	123.1918	-21.0130	-9.3477	-22.5081	-9.3946
**7m**	-8.8715	120.6809	-33.0147	-8.6818	-48.4946	-8.8715
**7m**	-8.6043	117.8894	-21.0437	-8.7636	-31.7588	-8.6043
**7m**	-8.3714	120.1070	-21.5628	-7.9919	-35.7030	-8.3714
**7m**	-8.1470	119.9670	-16.3607	-8.2047	-41.6722	-8.1470
**7m**	-8.0678	116.2293	-23.4112	-7.9267	-49.6466	-8.0678
**VX809**	-8.7534	0.5060	-31.5240	-11.3629	-59.9973	-8.7534
**VX809**	-8.6601	4.0513	-25.4632	-9.2755	-41.7578	-8.6601
**VX809**	-8.5667	10.5073	-27.5585	-9.2885	-50.9956	-8.5667
**VX809**	-8.4386	7.0309	-27.4009	-10.1334	-46.6416	-8.4386
**VX809**	-8.2508	9.4274	-27.4480	-9.3830	-30.5361	-8.2508
**VX809**	-8.0274	2.8064	-31.0662	-12.9882	-49.5065	-8.0274
**VX809**	-7.9213	3.5523	-32.9489	-10.8953	-48.5747	-7.9213

**VX809**	-7.6858	3.8581	-48.0437	-11.1632	-44.3771	-7.6858
**VX809**	-7.6615	7.3910	-31.7413	-11.8847	-37.2209	-7.6615
**VX809**	-7.5336	4.8728	-34.3204	-10.2162	-38.0646	-7.5336
**VX445**	-8.8879	-11.4383	-22.1244	-7.4005	-37.4239	-8.8879
**VX445**	-8.6380	0.0770	-19.2888	-7.9345	-13.6260	-8.6380
**VX445**	-8.2631	8.3296	-15.3546	-7.0670	-29.4215	-8.2631
**VX445**	-8.0541	-10.6323	-27.2839	-8.1029	-37.0600	-8.0541
**VX445**	-8.0460	14.5755	-30.9905	-8.1649	-19.9075	-8.0460
**VX445**	-8.0439	-0.7737	-26.1166	-7.7290	-37.7692	-8.0439
**VX445**	-8.0269	-2.5834	-26.1817	-7.3417	-15.0698	-8.0269
**VX445**	-7.9643	14.4722	-17.3904	-7.6905	-14.5522	-7.9643
**VX445**	-7.4496	36.0684	-20.7688	-7.3530	-26.0562	-7.4496
**VX445**	-7.3970	-10.1096	-27.4240	-8.6182	-34.4424	-7.3970
**VX661**	-9.7202	146.6608	-27.9352	-9.3063	-45.3188	-9.7202
**VX661**	-9.2703	147.3472	-41.3997	-9.8668	-45.8342	-9.2703
**VX661**	-8.9756	148.8089	-28.1827	-9.2730	-38.5385	-8.9756
**VX661**	-8.9596	145.6962	-27.9636	-8.9126	-43.4190	-8.9596
**VX661**	-8.8838	150.9577	-39.9014	-9.6556	-48.2573	-8.8838
**VX661**	-8.6724	152.3157	-34.5883	-9.1694	-43.5967	-8.6724
**VX661**	-8.5945	156.8173	-33.7225	-9.5907	-46.9538	-8.5945
**VX661**	-8.2392	150.3769	-27.9199	-9.1352	-38.6396	-8.2392
**VX661**	-8.0958	151.7892	-30.4774	-8.8291	-37.3454	-8.0958
**VX661**	-7.9088	150.6406	-26.6473	-8.5276	-35.8240	-7.9088
**VX770**	-7.8347	-33.9518	-17.3765	-8.0524	-3.3834	-7.8347
**VX770**	-7.0203	-34.2862	-21.8797	-8.1031	-5.9308	-7.0203
**VX770**	-6.0445	-47.5928	-21.2219	-7.9032	-17.9329	-6.0445
**VX770**	-5.8875	-48.1808	-21.8978	-9.7227	-19.8896	-5.8875
**VX770**	-5.7702	-50.7476	-30.4604	-8.0315	-18.0749	-5.7702
**VX770**	-5.3705	-62.9103	-18.8723	-9.0320	-21.2336	-5.3705
**VX770**	-5.3632	-64.4498	-17.8983	-8.3774	-19.6438	-5.3632
**VX770**	-5.2586	-64.6745	-15.4652	-8.2368	-17.4958	-5.2586
**VX770**	-5.2044	7.8535	-20.2484	-7.8782	25.8940	-5.2044
**VX770**	-5.1928	-60.1908	-20.1268	-8.4886	-18.3459	-5.1928

Table S3. Ten top scored docking positioning at the PDB code 8EIQ of the herein investigated hybrids **2a**, **7a**, **7m** and of the reference CFTR modulators **VX809**, **VX661** and **VX445**. The predicted ΔG value of each protein-ligand complex has been reported, as calculated in terms of final scoring function (S, as Kcal/mol).

Compound	S	E_conf	E_place	E_score1	E_refine	E_score2
**2a**	-8.3534	111.5993	-16.3119	-7.5907	-41.5406	-8.3534
**2a**	-8.2510	111.5280	-23.0820	-8.4811	-44.2405	-8.2510
**2a**	-8.2464	116.2800	-23.4254	-7.7754	-24.6866	-8.2464
**2a**	-8.0835	130.9052	-29.5426	-8.4959	7.4078	-8.0835
**2a**	-8.0270	114.0715	-34.0841	-8.1648	-44.2953	-8.0270
**2a**	-7.9737	123.2587	-28.1440	-8.2535	-23.4828	-7.9737
**2a**	-7.8299	109.9773	-29.4982	-7.9108	-39.1331	-7.8299

**2a**	-7.7772	112.0942	-21.5021	-7.4838	-30.2245	-7.7772
**2a**	-7.6574	111.6609	-34.8657	-7.5754	-45.2768	-7.6574
**2a**	-7.5278	109.2766	-29.3082	-8.0900	-41.5135	-7.5278
**7a**	-8.5864	111.6986	-19.6166	-8.4301	-38.3909	-8.5864
**7a**	-8.5337	117.9189	-24.3834	-7.9638	-12.3044	-8.5337
**7a**	-8.4784	108.7375	-22.2785	-8.9836	-34.5096	-8.4784
**7a**	-8.3238	110.5111	-20.6715	-9.0377	-32.9410	-8.3238
**7a**	-8.2339	108.4298	-30.4429	-8.6403	-41.1974	-8.2339
**7a**	-8.2304	107.4838	-30.5902	-8.4808	-48.6479	-8.2304
**7a**	-8.2076	106.3604	-33.8590	-8.0271	-44.9589	-8.2076
**7a**	-8.1941	106.1082	-17.3324	-8.1055	-32.2960	-8.1941
**7a**	-7.9427	114.3804	-24.8996	-8.3510	-22.4051	-7.9427
**7a**	-7.8847	104.8456	-32.1567	-8.4561	-44.4188	-7.8847
**7m**	-8.8892	114.9206	-22.2109	-8.2412	-37.0986	-8.8892
**7m**	-8.7612	118.6234	-25.7998	-9.2205	-48.4386	-8.7612
**7m**	-8.6524	115.9813	-20.9816	-7.9018	-44.3264	-8.6524
**7m**	-8.5723	126.6447	-31.3310	-9.4196	-2.9413	-8.5723
**7m**	-8.5250	119.3560	-30.7696	-9.5449	-45.7629	-8.5250
**7m**	-8.4863	115.9541	-30.6398	-9.5423	-47.6476	-8.4863
**7m**	-8.4188	118.3709	-31.1672	-7.8734	-45.2175	-8.4188
**7m**	-8.2799	118.8927	-35.1535	-8.5589	-39.5497	-8.2799
**7m**	-7.9816	120.7942	-28.5167	-7.9869	-41.9371	-7.9816
**7m**	-7.9016	117.7015	-30.4226	-7.8978	-44.8099	-7.9016
**VX809**	-9.0052	2.2213	-22.5744	-9.1115	-42.8242	-9.0052
**VX809**	-8.6360	1.0109	-21.6897	-9.2643	-47.0053	-8.6360
**VX809**	-8.1825	3.2027	-26.6370	-10.4513	-39.0939	-8.1825
**VX809**	-8.1520	0.4786	-30.8486	-9.6245	-43.6163	-8.1520
**VX809**	-8.1012	2.3734	-30.9284	-8.4550	-39.7959	-8.1012
**VX809**	-8.0947	1.9820	-33.7781	-8.0779	-41.0626	-8.0947
**VX809**	-7.7879	3.2906	-26.0511	-9.4302	-39.7564	-7.7879
**VX809**	-7.6487	8.6508	-20.1510	-8.0931	-26.9189	-7.6487
**VX809**	-7.6227	2.6937	-29.6413	-8.3318	-41.8047	-7.6227
**VX809**	-7.3294	1.2867	-32.2579	-10.4705	-30.3578	-7.3294
**VX445**	-9.5207	0.0342	-32.3420	-9.0708	-33.0028	-9.5207
**VX445**	-8.9537	-4.1180	-13.9611	-9.2306	-32.9748	-8.9537
**VX445**	-8.3979	-1.2488	-24.5129	-9.4438	-32.1247	-8.3979
**VX445**	-8.0046	-5.2436	-33.4995	-9.8872	-36.5432	-8.0046
**VX445**	-7.9867	-11.3411	-29.3282	-9.3418	-28.5844	-7.9867
**VX445**	-7.9020	-6.4645	-25.1217	-9.6491	-34.7645	-7.9020
**VX445**	-7.8158	-13.0721	-28.5840	-9.2695	-40.7140	-7.8158
**VX445**	-7.7172	-10.0348	-30.3656	-10.0866	-29.3394	-7.7172
**VX445**	-7.4673	-15.5061	-25.7903	-8.7002	-34.3133	-7.4673
**VX445**	-7.4092	-15.0419	-26.6748	-8.7892	-35.2276	-7.4092
**VX661**	-9.9823	150.1244	-40.2205	-10.9117	-45.3552	-9.9823
**VX661**	-9.7939	146.0527	-38.7318	-8.3753	-43.7035	-9.7939
**VX661**	-8.8051	147.8234	-44.9402	-9.7806	-42.0440	-8.8051

**VX661**	-8.7077	151.3457	-43.8284	-12.2488	-43.6721	-8.7077
**VX661**	-8.4495	148.1792	-47.2124	-11.3354	-43.9642	-8.4495
**VX661**	-8.4122	151.4115	-26.0129	-8.3495	-34.9538	-8.4122
**VX661**	-8.3407	144.5504	-33.5227	-9.3875	-41.6859	-8.3407
**VX661**	-8.1902	147.4272	-45.3528	-8.4205	-43.0460	-8.1902
**VX661**	-8.1781	153.5230	-31.7654	-8.2606	-41.7077	-8.1781
**VX661**	-8.0712	149.3826	-42.7571	-8.8094	-41.3579	-8.0712
**VX770**	-7.5505	-46.9035	-22.9151	-8.0302	-20.9402	-7.5505
**VX770**	-7.1309	-51.1266	-24.9403	-8.7437	-18.5165	-7.1309
**VX770**	-6.5369	-49.9515	-19.3523	-9.5596	-2.6425	-6.5369
**VX770**	-6.2985	-63.0451	-21.3399	-7.8532	-27.7993	-6.2985
**VX770**	-6.2882	-49.1486	-24.8200	-8.8010	-0.1246	-6.2882
**VX770**	-6.2698	-63.6828	-22.9188	-8.1095	-19.7896	-6.2698
**VX770**	-5.6648	-64.4831	-25.3537	-7.9872	-27.3219	-5.6648
**VX770**	-5.6163	-66.9415	-21.9140	-8.5708	-24.6298	-5.6163
**VX770**	-5.6062	-63.0726	-27.4657	-8.5783	-23.4933	-5.6062
**VX770**	-5.3798	-65.7533	-32.7362	-8.1598	-27.1794	-5.3798

Table S4. Evaluation of WT and F508del CFTR protein expression in HEK-t cells whole-cell lysates after treatment with 3 μM VX661, 3 μM VX445 and 2 μM **2a**, 2μM **7m**, 2 μM **7a**. Quantification either of total protein (C + B bands) and maturation rate (C/(C+B) bands) was obtained analyzing the band intensity with the software imageJ. Retrieved values were normalized to the intensity of the protein actin used as housekeeper protein and to the expression value of untreated F508del protein. Data represent the mean ± sem (standard error of the mean). For each condition, samples were run at least in quadruplicate. Comparisons of values with those of control, untreated samples were made with the Dunnett's test. Probability p values are reported. Asterisks indicate a significant difference ($p < 0.05$).

**WT CFTR**	**DMSO (Control)**	**VX661**	**VX445**	**2a**	**7m**	**7a**
**C+B**	1.00 ± 0.05	0.97 ± 0.09<br>P = 0.238	0.94± 0.05<br>P = 0.314	1.01± 0.04<br>P = 0.244	1.01± 0.07<br>P = 0.741	0.98 ± 0.07<br>P = 0.463
**C/(C +B)**	1.00 ± 0.01	0.99 ± 0.03<br>P = 0.298	1.02± 0.03<br>P = 0.237	0.98 ± 0.11<br>P = 0.453	1.02 ± 0.13<br>P = 0.747	1.01 ± 0.12<br>P = 0.224
**F508del CFTR**	**DMSO (Control)**	**VX661**	**VX445**	**2a**	**7m**	**7a**
**C+B**	1.01± 0.07	2.61 ± 0.27<br>P = 0.004 (*)	3.11± 0.37<br>P = 0.024 (*)	2.09 ± 0.25<br>P = 0.016 (*)	2.37 ± 0.19<br>P = 0.017 (*)	2.48± 0.16<br>P = 0.014 (*)
**C/(C +B)**	1.00 ± 0.11	2.10 ± 0.08<br>P = 0.014 (*)	2.50± 0.11<br>P = 0.005 (*)	1.42 ± 0.14<br>P = 0.011 (*)	1.68 ± 0.13<br>P = 0.016(*)	1.87 ± 0.08<br>P = 0.007 (*)

Table S5. Evaluation of CFTR single domain expression in HEK-t cells whole-cell lysates after treatment with 3 μM VX661, 3 μM VX445 and 2 μM **2a**, 2μM **7m**, 2 μM **7a**. Quantification protein expression was obtained analyzing the band intensity with the software imageJ. Retrieved values were normalized to the intensity of the protein actin used as housekeeper protein and to the expression value of each untreated single domain. Data represent the mean ± sem (standard error of the mean). For each condition, samples were run at least in quadruplicate. Comparisons of values with those of control, untreated samples were made with the Dunnett's test. Probability p values are reported. Asterisks indicate a significant difference ($p < 0.05$).

CFTR domain	DMSO (Control)	VX661	VX445	2a	7m	7a
**MSD1**	1.00 ± 0.05	2.19 ± 0.17<br>P = 0.012(*)	1.03 ± 0.07<br>P = 0.508	1.04 ± 0.05<br>P = 0.69	1.04 ± 0.12<br>P = 0.42	1.06± 0.08<br>P = 0.32
**WT NBD1**	1.00 ± 0.12	1.00± 0.15<br>P = 0.235	1.02 ± 0.18<br>P = 0.443	1.03 ± 0.09<br>P = 0.629	0.99 ± 0.04<br>P = 0.354	0.98 ± 0.08<br>P = 0.472
**F508del NBD1**	1.00 ± 0.04	1.02 ± 0.05<br>P = 0.507	1.01± 0.13<br>P = 0.464	2.06 ± 0.24<br>P = 0.013 (*)	2.19 ± 0.15<br>P = 0.004 (*)	2.32 ± 0.32<br>P = 0.035 (*)
**R domain**	1.02 ± 0.08	1.06± 0.09<br>P = 0.238	1.01 ± 0.04<br>P = 0.437	1.05 ± 0.08<br>P = 0.345	1.00 ± 0.05<br>P = 0.352	1.02 ± 0.05<br>P = 0.412
**NBD2**	1.01 ± 0.03	0.99 ± 0.04<br>P = 0.243	0.97 ± 0.11<br>P = 0.296	1.01 ± 0.05<br>P = 0.472	1.03 ± 0.11<br>P = 0.296	1.04 ± 0.15<br>P = 0.348
**MSD2**	1.01 ± 0.06	1.03 ± 0.05<br>P = 0.116	2.51 ± 0.15<br>P = 0.002 (*)	0.98 ± 0.13<br>P = 0.506	0.98± 0.06<br>P = 0.116	0.97± 0.16<br>P = 0.822

Table S6. Evaluation of F508del NBD1 protein expression in HEK-t cells whole-cell lysates after treatment with the compounds under investigation and protein new synthesis blockage with cycloheximide. Quantification of protein expression was obtained analyzing the band intensity with the software imageJ. Retrieved values were normalized to the intensity of the protein actin used as housekeeper protein and to the value of F508del NBD1 protein expression at time 0. Data represent the mean ± sem (standard error of the mean). For each condition, samples were run at least in triplicate. Comparisons of values with those of control, untreated samples were made with the Dunnett's test. Probability p values are reported. Asterisks indicate a significant difference ($p < 0.05$).

time (hours)	DMSO (Control)	VX661	VX445	2a	7m	7a
**0**	1.00 ± 0.01	1.00 ± 0.02<br>P = 0.411	1.00 ± 0.04<br>P = 0.232	1.00 ± 0.08<br>P = 0.681	1.00 ± 0.06<br>P = 0.331	1.00 ± 0.03<br>P = 0.145
**1**	0.72 ± 0.02	0.73 ± 0.12<br>P = 0.356	0.75 ± 0.12<br>P = 0.342	0.87 ± 0.05<br>P = 0.291	0.89 ± 0.03<br>P = 0.059	0.93 ± 0.06<br>P = 0.023 (*)
**2**	0.63± 0.08	0.62 ± 0.13<br>P = 0.741	0.65 ± 0.07<br>P = 0.624	0.78 ± 0.08<br>P = 0.027	0.81 ± 0.04<br>P = 0.114	0.85 ± 0.04<br>P = 0.022 (*)
**4**	0.43 ± 0.04	0.45 ± 0.11<br>P = 0.407	0.42 ± 0.09<br>P = 0.562	0.63 ± 0.03<br>P = 0.049 (*)	0.67 ± 0.04<br>P = 0.049 (*)	0.70 ± 0.03<br>P = 0.235 (*)
**6**	0.23 ± 0.13	0.26 ± 0.08<br>P = 0.176	0.27 ± 0.05<br>P = 0.405	0.45± 0.06<br>P = 0.019 (*)	0.50 ± 0.12<br>P = 0.035 (*)	0.53 ± 0.01<br>P = 0.023 (*)
**8**	0.07 ± 0.06	0.09 ± 0.03<br>P = 0.416	0.14 ± 0.03<br>P = 0.233	0.34 ± 0.03<br>P = 0.518	0.40 ± 0.09<br>P = 0.044 (*)	0.41 ± 0.02<br>P = 0.021(*)

Table S7. Primary antibodies used to detect whole length WT and F508del CFTR and CFTR single domains used in this work. For each antibody it is indicated the vendor, the clonality, the diluition (in PBS tween20 + 5% albumin) used to perform the western blot experiments, the epitope (when available in the data sheet) and the CFTR domain detected in our experiments

Antibody identifier	Vendors	Clonality	Diluition	Epitope	Target identified:
MM13-4	Millipore	Mouse monoclonal	1:200	N-terminus; residues 25-36	Full-lenght CFTR and MSD1
L12B4	Millipore	Mouse monoclonal	1:200	Residues 386-412	WT/F508del NBD1
Clone # 13-1 MAB1660	R&D system	Mouse monoclonal	1:100	R domain	R domain
ABIN350208	Antibodies on line	Rabbit polyclonal	1:100	Residues1150–1200	MSD2
clone M3A7	Millipore	Mouse monoclonal	1:200	C-terminus; residues 1365-1395	NBD2
